# Tip-Growing Robots: Design, Theory, Application

**DOI:** 10.1109/tro.2025.3608701

**Published:** 2025-09-12

**Authors:** Shamsa Al Harthy, S.M.Hadi Sadati, Cédric Girerd, Sukjun Kim, Alessio Mondini, Zicong Wu, Brandon Saldarriaga, Carlo A. Seneci, Barbara Mazzolai, Tania K. Morimoto, Christos Bergeles

**Affiliations:** 1 School of Biomedical Engineering & Imaging Sciences, King’s College London, London SE1 7EU, UK.; 2 School of Engineering and Materials Science, Queen Mary, University of London, London, E1 4NS, UK.; 3 LIRMM, University of Montpellier, CNRS, Montpellier, France.; 4 Department of Mechanical and Aerospace Engineering, University of California, San Diego, La Jolla, CA 92093 USA.; 5 Bioinspired Soft Robotics Laboratory, Istituto Italiano di Tecnologia, 16163 Genova, Italy.

**Keywords:** Soft robot, eversion growing, vine robot, additive manufacturing growth, plant-inspired robot

## Abstract

Growing robots apically extend through material eversion or deposition at their tip. This endows them with unique capabilities such as follow the leader navigation, long-reach, inherent compliance, and large force delivery bandwidth. Tip-growing robots can therefore conform to sensitive, intricate, and difficult-to-access environments. This review paper categorizes, compares, and critically evaluates state-of-the-art growing robots with emphasis on their designs, fabrication processes, actuation and steering mechanisms, mechanics models, controllers, and applications. Finally, the paper discusses the main challenges that the research area still faces and proposes future directions.

## I. Introduction

**Figure 1.**
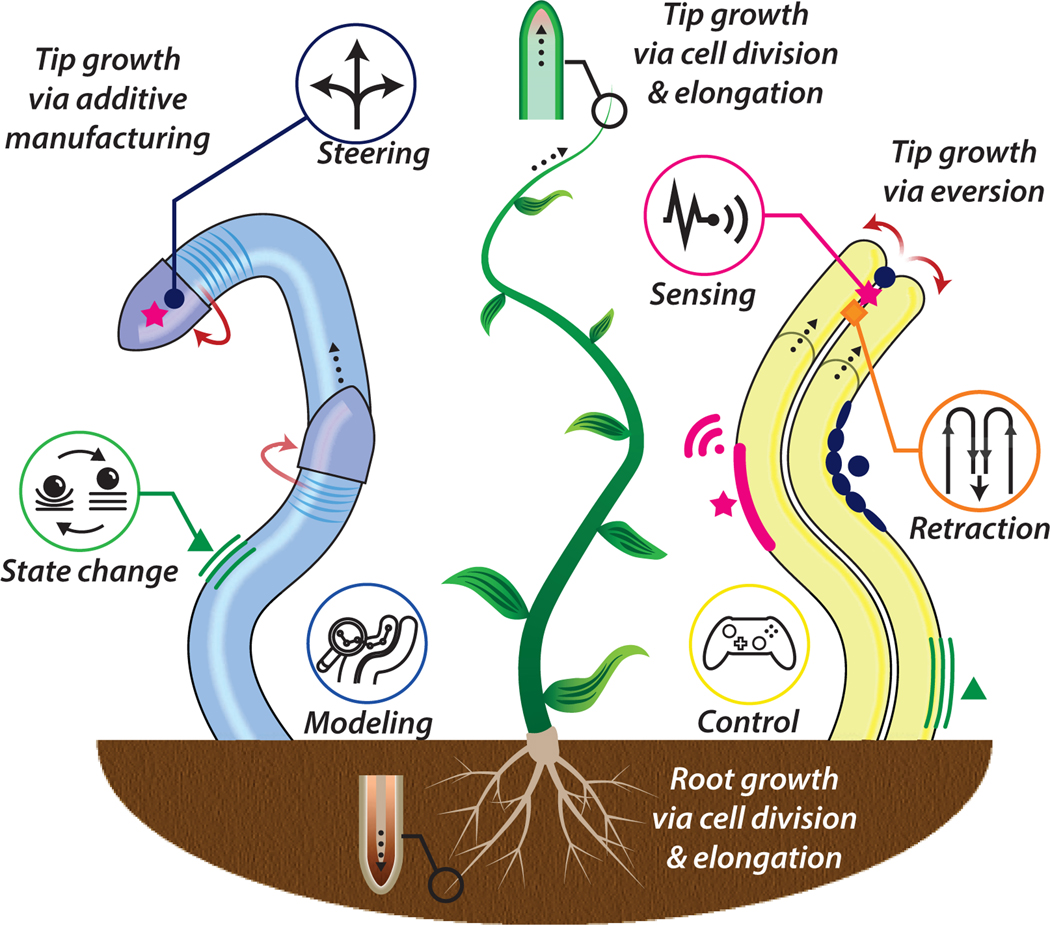
Representation of the two main tip-growing robot working principles proposed in the literature, i.e. material deposition via additive manufacturing (on the left) and pressure-driven eversion (on the right), and their bio-inspiration source, with a growing plant (middle).

DRAWING inspiration from the apical extension of plants’ roots and branches, tip-growing robots deploy through their environment by transporting material from their base to their tip. This new material then forms the body of the robot, onto which new transported material can be added. Navigation via apical extension enables the robot tip to advance while limiting the relative translation between the trailing body and the surrounding environment, making these robots attractive for deployment within sensitive and difficult-to-reach sites. To date, there have been two main working principles for bio-inspired, tip-growing robots: (a) pressure-driven eversion, and (b) material deposition via additive manufacturing, both represented in Figure 1.

Pressure-driven eversion leads to tip elongation through the unfolding and outward rolling of material stored within the robot body. This mechanism emulates eversion in certain animal species, such as the octopus retina, which turns inside-out when blinking to optimize vision [1], the extension and retraction of snail eyes in response to light [2], and the eversion of the proboscis during muscle contraction in spine-headed worms [3]. Eversion-based systems originated in the piping industry as early as 1977, for the renewal and repair of pipelines [4], and later emerged for vascular catheterization [5], endoscopy [6], [7], and colonoscopy [8]. The first eversion robot, i.e. a mechanism with built-in actuation, sensing, and low-level control, was introduced in 2006 by Mishima *et al.* [9] in the context of “SlimeScope”, a pneumatically expandable arm that navigated through rubble for search and rescue operations. Eversion growing robots were further developed by Tugokashi *et al.* [10] into an “active hose”, a multi-degree of freedom steerable eversion growing robot with high flexibility and low external friction. More recently, Hawkes *et al.* [11] reinvigorated research on eversion growing robots, drawing inspiration from prior efforts and showcasing a wealth of physically intelligent behaviors.

Many additive manufacturing growing robots use principles of traditional Fused Deposition Modeling (FDM) printing. Layer-by-layer deposition of fiber-based filament, fed from a spool at the base to the growing zone at the tip, was introduced by Sadeghi *et al.* [12] as a growth mechanism to imitate root growth and its ability to penetrate soil. Later, a FDM-based filament heating and plotting approach was introduced in [13] to achieve adhesion between layers while allowing the structure to bend. Further advances have facilitated passive morphological adaptation [14] and coiling motions [15]. Robots that grow via additive manufacturing can modify their shapes by tuning the printing parameters, may self-support their own weight, and demonstrate soft bending capabilities.

The literature review in [16] provided the first definition of growing robots as “a robotic entity that modifies its body structure by the incremental addition of material”, including cell-, molecule- or plant-inspired modular growing robots and organism-inspired growing robots (such as tip-growing robots). Comprehensive reviews of tip-growing robots were provided in [17], and [18], covering growth by eversion, and by additive manufacturing, respectively. Over 100 new papers have been published since then, detailing new designs, fabrication, actuation, sensing, modeling, and control approaches. Our manuscript revisits the research landscape to take a deeper look into the contemporary state-of-the-art, consolidating existing research and identifying trends and gaps in the field.

Our method of study was as follows. First, we defined keywords relating to the topic, e.g. “eversion growing”, “additive growth”, “additive manufacturing”, “soft growing”, “growing robot” and “vine robot”, which were entered into academic databases, including “Google Scholar” and “IEEEXplore”, to guide the search, in addition to key academic journals and conference proceedings. The forward and backward citations of each identified publication were tracked to ensure no relevant manuscript was missed. We limited the scope of this paper to the predominant approaches to tip-growth, i.e. everting and additive-manufacturing robots, illustrated in Figure 1. Other tip-growing robots, such as chain-block [19] and tape-measure [20] designs were excluded. Publications related to eversion outside the context of tip-growth, such as toroidal eversion robots, e.g. [21], were also excluded. The search led to 158 relevant manuscripts that are herein categorized, compared and critically assessed. We additionally contribute the creation and maintenance of an online public resource that provides details on the existing tip-growing robots^1^.

In the rest of this paper, Sec. II provides an overview of growing robot design, showing the different growth mechanisms that have been deployed, and the materials and features used in their fabrication. Sec. III highlights the steering, state-change, and retraction research, and Sec. IV details the work on perception and functionalization via sensor and tool integration. Sec. V describes the modeling and Sec. VI discusses the control approaches. Sec. VII showcases the application domains of growing robots. Finally, Sec. VIII summarizes the review and highlights gaps and future directions.

## II. Working Principle and Fabrication

**Figure 2.**
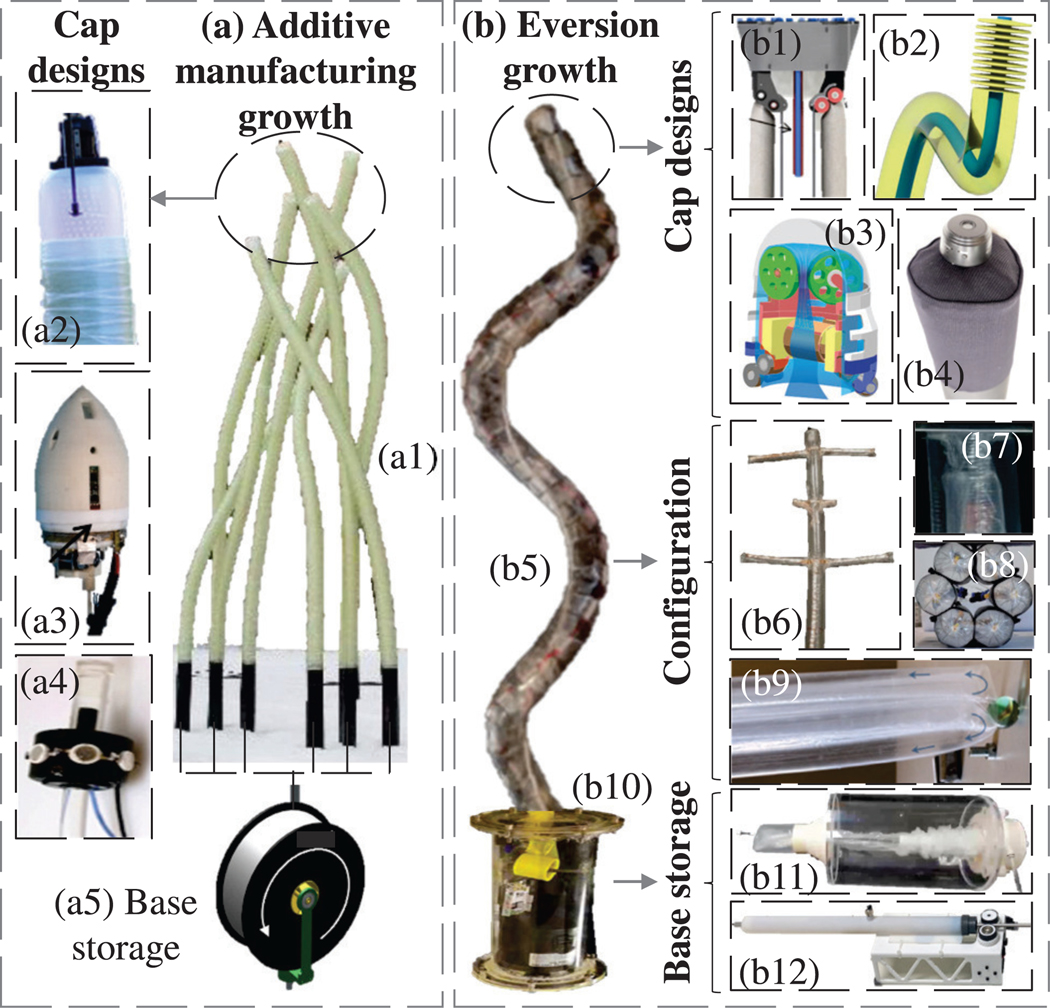
Design overview of tip-growing robots. (**a-left**) Additive-manufacturing robots [16] grow via (**a1**, **a2**) weaving [39], (**a3**) 3D-printing [15] or (**a4**) polymerization [40]. They rely on material deposition at the tip, typically with (**a5**) filament storage at the base [15]. (**b-right**) Eversion robots [11] grow by unfolding from the tip as pressure is applied. They use different cap designs to store eversion material, via (**b1**) a spool [41] or (**b2**) tip-material scrunching [29], or to integrate tools and sensors into the robot tip, via (**b3**) rigid outer caps [42] or (**b4**) soft fabric caps [43]. Different eversion robot configurations have been deployed, such as (**b5**) single-cavity, single-path straight or helical configuration [11], [33], (**b6**) branching of the robot body [33], (**b7**) nested bi-cavity structure [34], (**b8**) modular vine structure [26], or (**b9**) growing through bending of inflated tubes [36]. Material storage in eversion robots is achieved via (**b10**) spooling [11] or (**b11**) material folding at the base [24], or (**b12**) straight material storage in a long tank [44].

This section presents the working principles, design landscape, and materials used for developing tip-growing robots. Figure 2 illustrates the main growing mechanisms.

### A. Eversion-Based Growth

### 1) Concept and Working Principle:

This class of robots navigates their environment through pressure-driven eversion, whereby they unroll and deploy from the tip as internal pressure is applied [11]. The base of the robot remains in a fixed position with respect to the environment, and only the tip undergoes relative motion, reducing friction from body translation in the environment.

The everting body material is typically stored at the robot base, on a motorized spool [11]. However, this approach limits the use of the central robot lumen as a working channel. An alternative approach stored the inverted (tail) material straight, providing an open lumen to pass tools [22]. As the tail material translates at twice the speed of the internal components, friction between the tail and components limited growth length, which was further restricted by the pressurization chamber length. More recently, origami-inspired material scrunching (or gathering and folding) was introduced, where the tail material could be scrunched at the base [23]–[28], or at the tip [29], enabling both length-independent material storage and a continuous working channel. While eversion robots were generally pneumatically actuated, hydraulic actuation with more viscous working fluids had been explored for specific benefits, such as exhibiting buoyancy forces for underwater operation [30], achieving higher actuation forces [31], reducing buckling by increasing contact forces with the surface [32], and improving safety in surgical interventions [22].

Different robot architectures and configurations have been explored, with the most common being a single-cavity, single-path, non-modular robot. Helical structures have also been utilized to enable shape reconfiguration for deployable structures, such as antennas [33] and wearable haptics [23]. Recently, modular eversion robots were developed, comprising two concentric or parallel eversion tubes that grew independently as bi-cavity structures to improve grasping [34], [35]. Multiple tubes could be interconnected to grow simultaneously, improving retraction [36]. Another modular design proposed in [26], featured a sleeve-like main robot body, with smaller, circularly arranged eversion robots growing inside the main sleeve to provide an accessible working channel.

Beyond single-path everting robots, multiple branching configurations have been proposed to improve navigation. In [33], [37], pre-formed branches everting and lengthening during pressurization were used. These branches could be crumpled and stored at the base to prevent jamming [37], with length controlled by internal tendons [33]. On-demand branching via heat cutting, see [38], may allow multiple branches to operate simultaneously and to support the main body from buckling.

### 2) Materials, Fabrication and Trade-offs:

The performance of eversion robots highly depends on dimensions, tube thickness and pressure requirements. Larger robots are easier to deploy but require considerations like wireless communication, material jamming prevention, and faster inflation and deflation (e.g. centrifugal fans at the base) [45].

Smaller prototypes, currently with diameters as small as 2 mm [46], face challenges in achieving high aspect ratios due to fabrication, friction, and operational requirements. Everting tubes are often made by overlapping two material layers to create a seam. Thicker weld lines, while strengthening bonds, reduce flexibility, especially in miniaturized robots, where the seam width forms a larger ratio of the tube circumference.

It has been shown that vine robots without working channels that are scaled radially with respect to each other require the same pressure to grow [29]. However, this is not the case for vine robots that include a working channel. Indeed, the friction force between the working channel and the deploying tail, which is proportional to the working channel diameter, must be overcome by the growth force, which is proportional to the square of the robot diameter [29]. This means that a larger pressure must be supplied to compensate for the forces unbalance. Since the growth pressure must remain below the burst pressure, this restricts the robot’s growth length and down-scaling. Stronger materials or thicker tubes can increase the burst pressures, but reduce flexibility [47].

**Figure 3.**
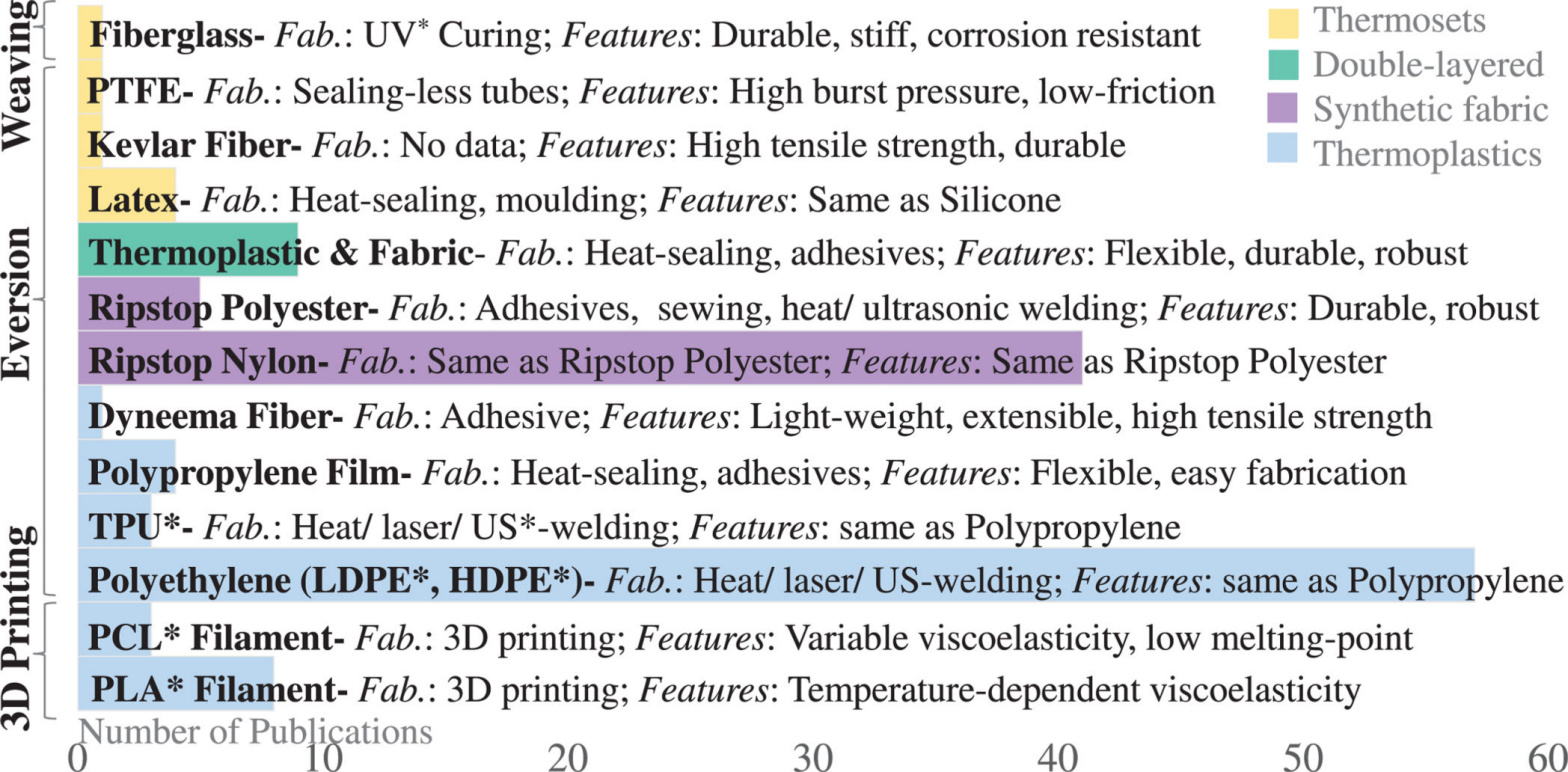
Growing robot material range, fabrication (Fab.) method, main features, and application frequency in literature.

Research into materials, structural designs, and fabrication techniques that overcome these limitations is required. The materials currently used in everting robots are characterized by their high compliance, low hysteresis, flexibility and, in most cases, inextensibility. Inextensible materials ensure that the pressure applied to evert the tip does not cause radial expansion or longitudinal stretching of the robot body, enabling shape change without requiring high energy [17]. Examples of such materials include thermoplastics, synthetic fabrics and thermosets, as detailed in Supplementary-Table I and Figure 3.

Thermoplastics are the most commonly used material due to their low cost and availability off-the-shelf in various diameters and lengths as seam-free, layflat tubing. Tubular everting structures can be manufactured in-house via heat-sealing [22], [31], [48], laser-welding [37], [49], or ultrasonic-welding [50]. Low Density Polyethylene (LDPE) is popular for its high tensile strength, flexibility, and ease of prototyping, but tends to retain its shape when stored on a reel, causing undesired curves upon inflation [51]. HDPE is an alternative, offering greater tear-resistance [17], [52] and more consistent growth due to its robustness to eversion pressure and tube diameter [53]. TPU and polypropylene exhibit higher strain limits than LDPE, as in [37], [48] and [31].

Synthetic fabrics, such as nylon and polyester, are highly compliant, flexible and robust. Coating nylon fabrics with silicone or TPU [54] can make the fabric airtight and waterproof. The fabric can be sealed into a tube using silicone glue (for silicone-coated fabrics), natural rubber adhesive (e.g. polyurethane), adhesive tape, or, for TPU-coated fabrics, heat-sealing [22] or ultrasonic-welding [50], [55], [56]. Ripstop fabric makes the tubular everting structures more durable and tear-resistant [57]. In particular, the high tensile strength of Nylon [58] and the high tear-resistance of polyester make them popular choices [41]. Polyester composites with reinforcement fiber mesh have been used in material scrunching [29], offering compact storage while enhancing tear resistance, tensile strength, and burst pressure. Double-layered tubes have been proposed, with inner LDPE layers providing an airtight seal and outer nylon layers enhancing durability [59], [60].

An alternative material class, hyperelastic thermosets like latex, was initially found to erode quickly at high temperatures, burst easily, and have limited repeatability due to their viscoelastic properties [61], [62]. However, recent work [63] demonstrated shape-locking possibilities, control of robot diameter at various locations through bulging, and reduction in tail tension, which lowered the robot’s tendency to buckle and improved its retraction capabilities.

Finally, recent designs have employed inhomogeneous materials or structures to create imbalances that enable steering or maintain tools at the tip [57], [64]. For example, the lower half can be fabricated with a more elastic material to facilitate directional steering via differential stretching [64].

### B. Additive Manufacturing-Based Growth

#### 1) Concept and Working Principle:

Additive growing robots apically extend by building their bodies *in situ* at the tip. To date, two main approaches have been proposed: FDM-inspired growth and photopolymerization. In FDM-inspired growing robots, the process involves feeding filament through a nozzle at the robot’s tip, where it is extruded and deposited in successive layers. Each layer bonds with the previous one, allowing the structure to grow and extend in a controlled, layer-by-layer manner. This method can be achieved using fiber-based filament fed from a spool at the base to the growing tip [12], or by equipping the growing head with a miniaturized 3D printer that pulls thermoplastic filament from a spool at the base, feeding it into a heating channel at the robot head. There, the thermoplastic filament is softened and extruded through a nozzle in a circular profile. Successive layers are formed, enabling the robot’s apical growth, similar to that of plants. Two motors at the head control filament extrusion (feeding) and head rotation (plotting), and together with the extrusion temperature, they enable structured material addition [13].

Additionally, the systems can actively adjust the material’s viscoelastic properties, allowing them to achieve sufficient rigidity required for applications like burrowing [13]. Their intrinsic state change properties can be exploited for obstacle avoidance and contact-assisted steering [14].

An alternative approach is photopolymerization [39], where a fiber-winding system uses soft fiberglass and Ultra-Violet (UV)-curable resin for fabrication. The system functioned by sliding and anchoring the robotic head to a reversibly inflatable mandrel to lock it in place. The head then weaved the fiber-reinforced composite along the exterior surface of the mandrel. The diameter of the resulting structure could be controlled by tuning the diameter of mandrel inflation. After the composites were cured, the silicone tube was deflated, leaving behind the composite structure. The robot could be steered before weaving the subsequent segment, allowing for control of the tube geometries and patterns through the fabrication sequence. This mechanism could build self-supporting, potentially interwoven, structures, with use cases in building walls and bridges.

More recently, [40] introduced additive growth via local polymerization by supplying the system with a constant flow of a monomer solution and solidifying it at the tip, then uniformly illuminating the structure to enable growth from the tip. Thus, mechanical and physical properties can be regulated.

#### 2) Materials, Fabrication and Trade-offs:

In classic FDM printing, performance depends on motor speed, extrusion temperature and material properties, all affecting print quality [15]. These parameters apply to FDM growing robots, which require high growth rates, regular material deposition and strong layer adhesion to self-sustain their weight.

High extrusion temperatures improve layer adhesion but can make the filament too soft to support its own weight, causing collapse. Such temperatures also stress motors, reducing the lifespan. FDM-based robots have slow growth rates due to operational limits, like motor speeds and cooling time. Faster feeding speeds accelerate growth but reduce extrusion temperatures, weakening layer adhesion [15].

Material parameters, e.g. melting points, require optimization. Lower melting points reduce energy use, decrease motor stress due to lower operating temperatures, and improve adhesion. However, they also lead to longer cooling times due to reduced heat transfer with the environment, resulting in high friction between the robot and the structure due to the deposited material remaining in the soft state for longer, irregular material deposition, and unstable structures. Material stiffness can also be problematic when scaling the system, as filament passage through small curvature sections may require high forces from the feeding motors. In summary, the material must exhibit low friction and stiffness to enable smooth and reliable plotting [65], and should be stretchable to enable differential bending [15]. All FDM materials have temperature dependent viscoelastic properties, [65], allowing the tip to soften when encountering obstacles.

PLA has been widely used for FDM-based robots [13]–[15], [65]–[67] due to its well-understood properties and availability. It has a high melting point (150–180°C) [68], enabling quick cooling, hence regular deposition and low plotting friction [15], but the high stiffness of PLA can stress the system [65]. Polycaprolactone (PCL), with a lower melting point (60°C) and stiffness, is an alternative, but can cause uneven growth due to its stickiness and slow cooling [65]. PCL was functionalized in [69] by mixing it with sodium alginate (SA) to form a biodegradable and biocompatible PCL/SA composite filament that absorbs heavy metals. TPU, ABS and PVA are less suitable due to their high melting points (220240°C), stressing motors. PVA offers a water-soluble solution for retraction of additive manufacturing robots, but is stiff, causing high plotting friction and motor stress. TPU and ABS are more flexible but not biodegradable. The high elasticity of TPU aids in bending, but can lead to poor adhesion, risking structural collapse [15].

Classic FDM systems pull filament from the base, sequentially depositing and cooling melted layers. Other approaches, such as depositing the filament in a liquid state and cooling layers all at once, aim to speed up the process but are less energy efficient [70]. UV-curable composites, like fiberglass-resins, have been explored for interwoven structures [39], producing durable, stiff, weather-resistant structures. In [40], a PDMS-PEA monomer underwent photopolymerization, offering tuneable mechanical properties (e.g. stiffness) via chemical adjustments (molar ratio and concentration). Supplementary-Table I and Figure 3 summarize materials and fabrication.

## III. Steering, State Change, and Retraction

Actuators and mechanisms have been integrated to enable functionalities in addition to growing, such as steering, state-change (including stiffening of the robot body), and retraction. See Table I (subscript) for the acronyms used in this Section.

### A. Steering

The steering mechanisms of tip-growing robots can vary widely between the two classes. In additive manufacturing-based robots, the same mechanism that drives apical extension is used for steering, i.e. the growth orientation is changed by controlling the material deposition parameters. In comparison, eversion robots usually require dedicated structural modifications (e.g. pre-defined morphology, pouch actuation) or additional hardware (e.g. tendons, latches) for steering. If such solutions are not integrated into the eversion robot systems, they can rely on their inherent compliance for navigation in cluttered environments via self- or wall-interactions. Passive and active steering solutions have been proposed for the two classes, as in Supplementary-Figure 1 and Table I.

#### 1) Passive and Environment-Assisted Steering:

Passive steering does not rely on active control of actuators and sensors. It includes pre-defined morphology, which uses the robot’s inherent design to guide movement, contact-assisted steering, which leverages environmental interactions to steer the body, and material response to external stimuli.

- **Pre-defined Morphology:** Steering can be achieved by preforming the eversion robot body prior to deployment, e.g. using adhesive tape [50], [71] or fastening rigid connectors [50] to create bends, soldering or welding bends onto the material [50], or creating a pre-shaped tube [61], [72], [73]. This approach simplifies the system but is limited to known static environments.
- **Contact-Assisted Passive Steering:** While obstacles are usually seen as hindering robot motions, recent work has explored the use of obstacles to aid navigation. Growing robots can passively adapt their morphology upon contact with the surroundings. For eversion robots, this can be in the form of self-interactions in highly constrained environments, or wall-contact, see [74]. Additive growing robots can also be passively steered using the ductile phase of the PLA at higher temperatures [14], or the inherent compliance of the material [40], to adjust the robot path as it encounters obstacles.

Contact-based steering simplifies the system as it does not require extra hardware. However, this approach can be less suitable for open environments or where multiple pathways exist, as the robot simply follows the path of least resistance.

- **Material Response to External Stimuli:** Material-level responsiveness allows steering in response to external stimuli without input from the central robot controller. For example, [75] used a low-boiling point fluid (Novec-7000), encased in pouches along the eversion robot body, to steer the robot in response to external stimuli (infrared light), which caused pressure changes. This method leverages principles of embodied intelligence for an instant steering response at a material level, but requires environments where external stimuli, like light, can be easily transmitted.

### 2) Active Steering:

Active steering enables a wider variety of shapes. Different mechanisms for distributed steering along the entire robot backbone or for localized articulation of the tip or body segments have been proposed.

#### a) Distributed Steering:

Methods that deform a significant length of the robot have been shown for eversion growth.

- **Tendon-Driven:** Tendon-driven steering features single or multiple tendons routed along the robot’s entire body, often inside guides spaced evenly along the body. Pulling the tendons causes contraction in the regions between the guides, allowing uniform deformation. Tendons have been placed either axially [76] to enable bending or helically around the robot to produce bending and torsion for helical shapes [77]–[79]. This method is repeatable, precise, and can enable reversible shape changes [55]. However, it may create friction forces with the environment, and the guides must withstand the eversion process at the robot’s tip.
- **Pneumatic Artificial Muscles (PAMs):** PAMs are inflatable contractile actuators that convert compressed gas to mechanical energy [80]. As PAMs locally contract the robot body over long lengths, they provide more uniform bending along the length compared to tendons and tubular guides. In addition, they are entirely soft and do not translate with respect to the environment [81]. PAMs have also been designed for hydraulic inflation of pouches [82], and could be engineered to elongate, as seen in inversive PAMs (iPAMs), which stretch when pressurized, offering additional capabilities.

One type of PAM is known as series PAMs (sPAMs) or pouch motors (SPMs), which consist of a tube with partial seals that form radial constraints (sPAMs) or flat surfaces (SPMs) along its width, repeated along its length. These tubes are attached along the robot’s pneumatic backbone. Upon inflation, the pouches balloon and contract the robot body [80], [83]–[86]. Internal attachment of the pouches has been shown to improve bending by 133% compared to external attachment [81], and adjusting the sPAM configuration (number of pouches and arrangement) directly impacts the achievable bending angles [87]. Another class of PAMs are fabric PAMs (fPAMs), which leverage the patterns in ripstop fabrics. Introducing a bias-cut in the fabric provides the actuator with elastic properties, compared to the inextensibility presented with straight cuts, i.e. when cutting along major thread lines [54]. The fabric bias permits fPAMs to simultaneously expand radially and contract laterally as they are pressurized [54]. As fPAMs use low-friction fabrics to create a single tube, they are easy to evert and demonstrate quicker response times than alternative PAMs [55]. IPAMs comprise a latex rubber tube that lengthens when pressurized, while fibers wrapped around the actuator limit its radial expansion [33]. The use of elastic material in iPAMs produces a non-linear stress-strain graph, complicating control [57]. Cylindrical PAMs (cPAMs) integrate cylindrical pouches directly onto the robot body, acting as the upper layer, which increases their robustness [55], [81]. Unlike other PAMs (e.g. sPAM), cPAMs are not constrained by folded material along the edges, enhancing their bending performance as they undergo significant cross-sectional deformations during pouch inflation [55].

- **Magnetic Steering:** Magnetic steering has been explored to reduce space requirements in growing robots. Davy *et al.* [88] proposed coating the LDPE robot body with a thin layer of Neodymium-Iron-Boron (NdFeB) magnetic powder-doped silicone, with steering controlled by an external magnet. This layer needed to be thin to avoid hampering the robot’s flexibility, but this limited the available magnetic volume. Alternatively, [89] functionalized a magneto-rheological fluid for fluidic actuation and steering, preserving both the robot’s flexibility and magnetic volume. Both approaches show potential for miniaturization but require the magnetic platform to be placed within very close proximity to the robot to generate a sufficient magnetic moment for steering.

#### b) Localized Steering:

Steering mechanisms that articulate specific/selected locations along a growing robot, e.g. in the form of a local sharp bend, are discussed here.

- **FDM-based Steering:** This class of robots achieves steering by tuning the amount of deposited material along the plotting circumference, which permits to change the growth orientation. FDM-like growing robots are actively steered by tuning the three printing parameters: (1) plotting speed, (2) filament feeding speed, and (3) extrusion temperature, to either control the viscoelastic properties or the material deposition rate and volume. Differential deposition was widely explored for steering such robots. This was implemented in [13], [97] by depositing a different number of layers on opposite sides of the robot (radially) to induce bending, but this is impractical as it results in slower growth rate (due to the need to frequently interrupt and reverse the deposition) and weaker structures [97]. A more effective steering strategy involves tuning the layer thickness while keeping the same number of deposited layers [97]. The layer thickness is controlled by varying the plotting velocity along the circumference. Specifically, when the plotting speed increases, the layer becomes thinner (steering direction), while in the opposite condition, the layer becomes thicker (opposite side of the steering direction). This behavior can be achieved by controlling the rotation speed as a function of the plotting angle using a sinusoidal law. This mechanism later evolved in [15] by redesigning the robotic FDM-tip to couple the control of the plotting speed and filament feeding speed, improving differential deposition and enabling a smaller steering radius. Together with the plotting speed and filament feeding speed, reducing the overall growth speed can be helpful in cluttered environments.
- **Weaving:-**based growing robots control curvature by steering the robotic head first, then winding the glass fibers in the position taken by the robotic head [39]. This method contrasts with differential deposition strategies in FDM-like robots, where curvature is induced by controlling the plotting and deposition parameters.
- **Latches:** Among the first proposed active steering mechanisms were mechanical latches, which retain robot material in their locked configurations. Upon applying pressure to external chambers around the eversion robot body, the latches snap and release stored material, thus lengthening the corresponding robot side [11]. This steering method is not reversible.
- **Magnetic Valves for PAMs:** Kubler *et al.* [91] proposed connecting cPAMs in parallel for independent pouch actuation of eversion robots, overcoming the curvature limitations of serially connected PAMs. Each pouch featured a 3D printed magnetic valve, controlled by an external magnetic field, enabling selective inflation for local bends while retaining the shape and providing greater degrees-of-freedom with few control inputs.
- **Internal-device Steering for Eversion Robots:** An alternative for localized steering in growing robots is the use of internal devices, such as the INCHIGRAB, which employs a linear actuator-anchoring module at the tip to steer the robot and propel it forward using inchworm-like motion along curved paths [98]. Other methods include steering-reeling devices that enable bending by rotating internal segments [95], integrating a continuum robot in the eversion robot body [94], or passing a wheeled robot through the working channel [27] to increase the growing robot’s degrees-of-freedom. Rigid links, tendons and twisted string actuators (TSAs) have been used for localized bending by shortening one side of an internal mechanism [48], [92], [93]. Magnetic tips enable high-curvature, tip-localized steering [28], but proximity issues and reduced magnetic volume in smaller magnets limit their effectiveness. Heat-welding mechanisms can be incorporated into internal-steering devices in order to construct bending structures in real-time, based on wall-contact or pre-programming [58], [96], [99], with length differences between welded and unwelded sides causing bending. While these internal devices enable large bending angles and tight curvatures, they typically require rigid components, limiting compliance and increasing size and weight.

### B. State Change for Shape Retention and Force Transmission

**Figure 4.**
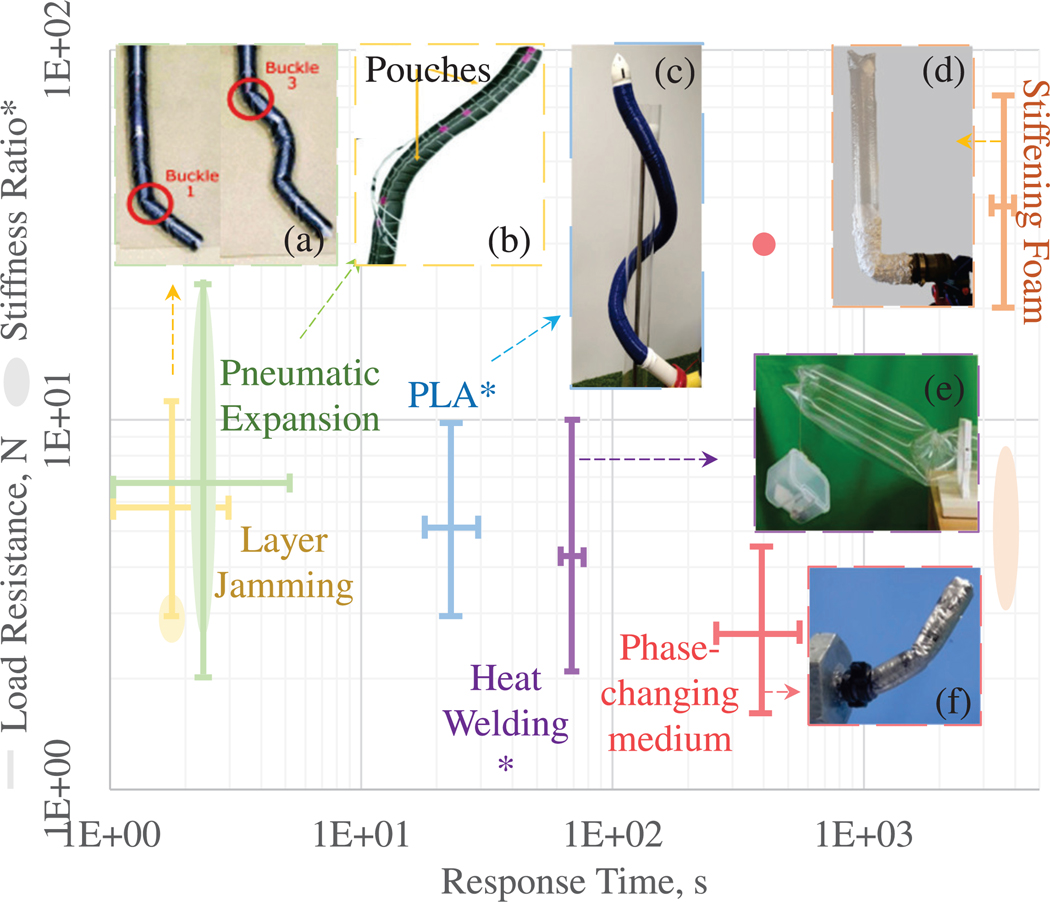
Controlling stiffness in growing robots through (**a**) layer jamming [102], (**b**) pneumatic expansion [81], (**c**) PLA (polylactic Acid) property change [15], (**d**) stiffening foam [103], (**e**) heat-welding [58], and (**f**) phase-changing alloy [104]. The graph indicates the relationship between load resistance (line), stiffness ratio (oval), and response time for all mechanisms. All data was retrieved and averaged from the respective papers. *No stiffness ratio data on heat-welding and PLA.

Growing robots are reconfigurable with infinite degrees-of-freedom. In constrained pathways, they achieve multiple bends by conforming to the environment. However, in open spaces, the limited number of actuators in growing robots makes it difficult to fully activate their degrees-of-freedom, unless external components are integrated. The inherent compliance of eversion growing robots affects tool stability, force exertion, and their tendency to buckle under high loads. Shape locking and stiffening mechanisms can control a robot’s state and structural deformation to achieve the required degrees-of-freedom while increasing force transmission. However, achieving state change in growing robots can be challenging due to their continuously changing morphology. The mechanisms discussed below are independent of steering and applied afterward, while steering-related state change mechanisms were addressed in the previous Section. Supplementary-Table II and Figure 4 compares the various state change methods.

Passive shape-locking was demonstrated with simple designs, such as loops enclosing the robot body at the desired locking point. In [100], [101], Velcro straps enclosed the tubular robot body, permitting multi-bend shape locking by controlling the location of the straps. Active shape-locking requires additional actuation, increasing complexity. This was demonstrated in [76], where shape-locking segments were passed through guide-tubes placed along the body of a tendon-driven robot (which evert independently of the main body), locking curves in position. Active shape locking is often coupled with variable stiffness mechanisms, enabling steering in a compliant state and shape retention in a rigid phase.

Modifying a structure’s geometry enables variable stiffness while maintaining material properties. Pneumatic actuators control stiffness [60], [81], [83] by tuning the relative pressures in the robot body and actuators. Lower actuator pressure and higher chamber pressure increased stiffness [81], improving trajectory tracking [83] and force exertion [62], [73], [84], [105] while maintaining a soft body during deployment. Pneumatic expansion has been used with tendon-driven steering [105], where air pressure and tendon tension were controlled to modulate stiffness. However, pneumatic expansion presented time delays due to the time taken to fill the pouches.

Layer jamming enables stiffness control by leveraging friction between two or more layers. Pouches containing layers could be arranged in series to form the robot body. By controlling the inflation pressure, the sequential jamming and unjamming of the pouches allows for stiffness control, enabling smaller tip deflection, shape retention [56], [102], [106] and higher force exertion [107] during the jammed state. Layer jamming also aided distributed steering by simultaneously stiffening one side and stretching the other, uniformly-wrinkled side, allowing it to bend and achieve tight curvatures [107]. Despite its simplicity, layer jamming had limited stiffness ratio (up to 2) [102] and introduced time delays when switching between states. The response time could be improved via direct pouch pressure control [56].

Instead of inducing structural changes, stiffness can be controlled by tuning material properties via temperature and state change using a phase-changing alloy. In [104], the alloy was used as the actuation fluid to evert the robot and served the secondary purpose of stiffening when solidified. The mechanism resisted higher forces than jamming-based methods and occupied less space within the robot body, but it did not allow for continued growth while in the stiff state.

A foam-based approach [103] involved spraying expanding polyurethane foam, which stiffened as it dried. The foam could passively maintain rigidity and structure, but suffered from a slow response time (approximately 1 hour) and may not be suitable for sensitive/fragile environments.

Finally, electroadhesive pads utilize field-induced stiffness change, and could be integrated into the robot body [35]. As electric fields were applied, the attraction forces of the pads increased, strengthening contact and adhesion, which, in turn, improved payload capabilities for grasping applications.

### C. Retraction Mechanisms

Eversion robots, due to their material compliance and unfolding mechanism, theoretically have the ability to reverse growth by inverting (folding inwards) at the tip to reduce their length (retraction). Successful retraction allows the robot to withdraw its body without causing damage. However, retraction becomes particularly challenging at longer lengths due to the robot’s tendency to buckle and collapse under retraction forces applied at the tail, limiting motion control and potentially applying undesired forces on the environment [108]. Therefore, in practice, buckling during retraction remains a major challenge. We note that some mechanisms intended for steering (e.g. mechanical latches in [11]) or shape locking (e.g. foam-based stiffening in [103]) can increase overall rigidity, hindering retraction and making growth irreversible.

One approach reduces buckling by integrating rigid reeling mechanisms directly at the robot tip instead of the base [42], [95], [108]. This mechanism artificially reduced the length of the robot, alleviating tail tension and expanding the conditions under which retraction occurs without buckling.

To stiffen the robot axially and pull the tail material at the base, Pi *et al.* [109] proposed integrating a flexible, incompressible tube between the robot body and tail. Moving the tube backward while maintaining tail tension prevented buckling. The system, however, experienced high friction between the tube and the robot body in curved paths.

Instead of relying on rigid elements, [32] used water as the working fluid for robot eversion, maintaining contact and friction with the ground during retraction to prevent buckling. However, this method increased weight and limited the robot to 2D environments with planar contact constraint.

Soft retraction mechanisms, such as inchworm-like motion, were explored in [98], [110], where the tail was pulled backwards by an internal module. Negating the need for additional hardware and complex control, the growing robot in [111] incorporated an internal central retraction tube; inflating this tube while the robot body was depressurized caused a sealing ring to move forward, automatically inverting and self-retracting the robot. Larger channels enabled more efficient retraction but increased jamming risks.

Another factor hindering retraction is the lateral shift eversion robots experience during growth, creating a bending moment. Wu *et al.* [36] demonstrated isovolumetric eversion by interconnecting multiple tubes, ensuring eversion along the central axis and reducing retraction forces.

As noted, research on retraction has mainly focused on eversion growing robots. Robots that grow via additive manufacturing lack an inherent retraction capability. As they are required to be pulled out of the environment, they can generate high forces against the surroundings, limiting their use to applications where reversible growth is unnecessary. These robots have been tested for deployable structures and environmental monitoring, where the absence of retraction can be advantageous for creating permanent structures. However, in compromised or sensitive environments, the lack of retraction may pose a limitation, as further detailed in Section VIII.

## IV. Environment and State Perception, and Robot Functionalization

Sensors and tools enhance the navigation, performance and usability of growing robots in confined spaces. A representative sample is depicted in Supplementary-Table III.

### A. Sensing and Perception

Various proprioceptive and extereoceptive sensors have been used within growing robots. For grouping purposes, we define proprioceptive sensors as those that record the state of the robot (e.g. position and orientation) via internal or external means. Exteroceptive sensors perceive the surrounding environment, providing information on the environmental conditions or responding to external stimuli. We expect neither sensor category to necessarily be linked to control feedback.

#### 1) Proprioceptive (Robot State) Sensing:

Proprioceptive sensors provide information on the robot’s position orientation to localize the tip, sense curvature, or estimate contact forces.

- **Tip Localization:** Watson *et al.* [112] integrated a fixed ring-shaped permanent magnet at the robot’s tip, complemented by an array of magneto-inductive sensors in the environment, to localize the tip position and orientation, albeit within limited range due to proximity constraints. This approach enabled tip tracking in surgical environments, where accurate state and position information is crucial. Similarly, optical markers have been used for testing purposes to estimate position changes [55], or for model verification, which could later inform model-based feedback position control [113].

More recently, Raines *et al.* [114] proposed an acoustic approach, detecting pressure and acoustic signals at the robot base to infer environmental changes, e.g. localizing the robot position when traveling through tunnels of varying sizes. This approach could be challenging to deploy in noisy environments, as it is harder to retrieve meaningful acoustic signals. An alternative, explored in [115], used sensorless tracking via model-based pressure regulation to link feeding pressures to eversion length and tip position, as discussed in Section V.

- **Curvature and Shape Sensing:** Accelerometers can be used to detect robot orientation with respect to gravity, guiding the growth through helical pathways, as seen in additive growing robots [15]. While accelerometers are highly accurate [116], they estimate strain from known kinematics, rather than measuring it directly [117]. Eversion-growing robots, with their non-linear deformation, make kinematic modeling more challenging, leading to potential sensing inaccuracies.

Instead, a contact switch array and IMU were deployed in the tip-mount in [117], providing direct strain measurements with real-time orientation feedback. In [116], IMUs were integrated onto flexible PCB sensor bands and distributed along the robot’s length, with the IMU orientations feeding into a model to estimate the 3D shape of the structure. However, these mechanisms relied on magnetometer IMUs, which have limited heading accuracy (i.e. when sensing shape and orientation) compared to accelerometer IMUs, and are susceptible to interference from external magnetic fields.

Alternatively, [118] employed optical wave-guides along the robot’s length, which provided bending information along the different segments and enabled local curvature tracking for grasping applications. Optical waveguides experience signal loss at higher bending curvatures due to compressive strain.

- **Force Sensing:** When encountering obstacles, growing robots exert axial forces at the tip and perpendicular forces along their length [117]. Direct force sensors are difficult to integrate into the robot itself due to the need to excessively bend with the robot during eversion, leading to non-linear deformations to the force sensor that reduce sensor accuracy and signal-to-noise ratios. When placed at the tip, the dynamic nature of eversion would result in high noise levels. Instead, the force sensor can be placed in a region less dynamically affected by eversion motion, e.g. by passing FBG-sensor fibers along a catheter to measure tip forces [46].

As another alternative, soft resistance sensors [119] have been used to detect contact forces from changes in curvature. This method, however, does not provide direct force measurements, and instead estimates the forces based on the difference between the curvature sensor measurements and the model-predicted curvature.

Pressure sensors integrated into the steering pockets of the robot offer another approach, providing direct feedback on contact forces. Forces applied to the pockets linearly increase internal pressure [120], which can be leveraged for obstacle detection with feedback control.

#### 2) Exteroceptive (Environmental State) Sensing:

Vision-based sensing, exemplified in works such as [9], [11], [30], [45], [80], [85], [86], uses tip-mounted cameras to provide direct and real-time visual feedback of the environment. Such sensors can be challenging to mount due to their weight, growth length constraints [11], [80], or bulky housing [9]. To address this, a more compact, tethered camera-mounting design was presented in [85]. Light and heat sensing mechanisms, as in [75], utilized a thermoresponsive liquid (photoabsorber) as working fluid in growing robots to initiate a sensing-steering control loop, steering the robot by contracting its body towards the direction of the source. Del Dottore *et al.* [15] demonstrated another approach using a sensorized tip with photoreceptors at the robot apex. By sensing light and wavelength, the growth orientation could be adjusted to implement plant-inspired behavior as phototropism and skototropism. Several sensorized tip-mount caps have also been developed, such as in the “plantoid” [13], [121] and the robot in [65], which incorporated temperature, humidity, chemical/water content sensors to monitor subsoil conditions.

### B. Robot Functionalization: Component Integration

The integration of the sensors and functional tools into growing robots involves various designs, focusing on tip-mount components or their distribution along the robot’s body. This includes cap designs, e.g. string-mounts, magnetic caps and soft caps, working channels or sensor distribution.

Both, eversion and additive growing robots are intrinsically tethered to the base for material feeding. In additive growing robots, most actuation components are brought to the tip (apart from the spooler at the base). The actuation components require high power, necessitating a tethered cap at the tip, which can simultaneously be used for sensing and functionalization purposes. In contrast, eversion robots retain axial actuation components (e.g. material, control board, pressure generator) at the base, allowing actuation and sensing to be separate modules, and enabling sensor distribution along the body or encapsulation in an untethered tip mount.

#### 1) Tip-mount Components:

Mounting sensors and tools at the tip offers direct and localized feedback during deployment, but is difficult as the new material added at the tip needs to be followed. Placing a rigid tool at the tip also increases the weight and diameter of the growing element, limiting maneuverability, slowing growth, and restricting access to apertures smaller than the tip diameter. Several cap designs have been featured in growing robots, further detailed for eversion robots in [42]. The mount can be positioned at the tip, within the pressurized area, or along a working channel.

- **Tethered Cap Design:** A tethered design, where the sensors were directly tied to a string running through the eversion robot body, was proposed in [11], [80], [122], [123]. As the string moved twice as fast as the tip, it had to be pulled back from the base. In such cases, the tail material could not be stored in a spool and the growth length was limited due to friction forces between the string and the tail material [42]. To mitigate this, Kim *et al.* [24] introduced an origami folding and feeding mechanism for tail material storage at the base. Alternatively, string management mechanisms such as a wire rewinding tool [9], and a zipper pocket mechanism that runs along the robot length [85] have been implemented. The latter, coupled with a rigid outer cap, held tools in place, though the lack of friction during retraction caused cap detachment.

Additive growing robots typically use tethered tip-mount caps due to high power consumption for material feeding and deposition, combining actuation and sensing [13], [15], [39].

- **Untethered Cap Designs:** To remove the constraints of a tether, several outer cap designs that are mechanically fixed onto the tip have been proposed. In [30], [124], a magnetic mount was placed inside the pressurized area to maintain its position at the tip during growth and retraction. However, weak magnetic forces may cause the outer part to detach. Combining previous designs, Jeong *et al.* [42] developed a tip mount with an outer cap for sensors, retraction device, and magnetic interlock. This design used an electromechanical roller-based design with a hook attaching the outer cap to the inner segment, preventing it from physically separating. At a larger scale, Heap *et al.* [45] introduced an umbrella-shaped tip mount that opens during growth, with sliding contacts to guide its motion, and closes during retraction. While these designs improve functionality, they may reduce growth speed, robot compliance, and increase weight. Heap *et al.* [110] presented an internal camera mount using ball bearings and a PTFE balls to reduce friction and weight, enabling the robot to navigate tighter gaps. Alternatives, like soft fabric caps [43], [64] and origami-inspired tool-feeding mechanisms [24], secure tools at the tip without relying on rigid components.
- **Tool and Sensor Delivery via a Working Channel** is an explored approach, though high robot internal pressures compress the channel, requiring pressure control during tool transmission. One design attaches the working channel tip to the base of the tail, moving with it until it reaches the tip of the robot at the end of the deployment [72]. This limits access to the robot tip at any time during deployment. A design that enables access to the tip at any time is possible if the working channel is inserted inside the robot tail. However, since the tail translates at twice the speed of the working channel, the working channel needs to be held back at the base, generating friction forces between the working channel and the tail. Solutions include duty-cycle controllers to align tool transmission with robot pressurization/depressurization cycles [22], [125] or constantly blowing air between the working channel and robot body to reduce drag [126]. Passing a semirigid channel along the robot body can also prevent airflow into the central core [111]. Structural designs, such as tip-scrunching [29] overcome working channel scaling and length limitations while allowing tool deployment during eversion. The modular design in [26] (see Section II) used pressure-driven eversion of sub-vines to push the main vine forward (instead of directly inflating it), decoupling inflation and working channel pressures. The latter remained at atmospheric level, enabling tool transmission.

#### 2) Component Distribution Along the Robot Body:

Distributing sensors along the robot’s length aids in localization, obstacle detection, and monitoring of the robot navigated path. Examples include distributing sensors along the body [116], [119], [120] or within branches [37]. Distributed sensing can be particularly useful for force and shape sensing. Embedding sensors in the flexible structure of eversion robots is challenging as the sensors would have to evert together with the robot body. However, there exists a stiffness mismatch between available sensors and the eversion robot body, which can easily intervene with the robot’s motion and functionality [116], [119]. To address this, sensors can be distributed by embedding them within the steering pouches [120] or integrating flexible sensors within individual pouches to allow for material wrinkling, harmonizing stiffness along the robot length [119].

## V. Modeling

Modeling growing robots is challenging due to their changing morphology, buckling tendencies, and non-linear dynamics. Supplementary-Figure 2 and Table II highlight the main approaches.

### A. Kinematics

Several kinematic models have been proposed for growing robots. The Pseudo Rigid Body (PRB) method, also known as joint-space representation, approximates continuum robot backbones using conventional rigid-body links and joints, such as a prismatic-revolute-prismatic configuration. This method was revised to accommodate changes in robot length by updating the number of joints and rigid links, thus modifying the equation of motion. [95]. These models have been used for design optimization [106], [139], [140], obstacle-aided path planning [71], [137], contact localization [117], shared control [138], and in open-source dynamic simulation software [136].

Alternatively, constant curvature models can predict motion through geometric constraints in actuators that are parallel to the backbone [79]. A kinematic model for an sPAM actuated growing robot, considering the effect of pressure on tip displacement was proposed in [137]. A similar kinematic model for robots that grow incrementally by means of additive manufacturing was proposed in [66], and further developed in [127] by considering configuration space motion to describe robot motion and define suboptimal 3D trajectories.

While PRB and constant curvature models can accurately describe the geometry of simple systems, they are unable to capture the complex highly nonlinear motions of slender soft systems. Therefore, variable-curvature models based on Cosserat rod theory [79], Piecewise Variable Curvature (PVC) method, i.e. concatenation of segments with variable curvature [145] and reduced order shape [22], [142] fitting by a shape function were introduced. For example, Blumenschein *et al.* [79] used variable curvature and geometric constraints formulations to develop a kinematic model for helically actuated pneumatic growing robots. Wang *et al.* [142] divided the system representation into a spatial curve for the robot’s geometry and a reduced-order kinematic approximation using piecewise cubic Bezier curves, enhancing the model’s positional accuracy. A similar approach was proposed by Allen *et. al* [113] based on a polynomial representation of the robot continuous curvature (here only bending angle).

### B. Quasi-Statics Models

The kinematic models mentioned only considered geometric parameters like robot radius and steering actuator locations. For everting robots, quasi-static models have been proposed to account for internal and external forces, such as internal pressure and external contact. These models are used to understand growth, retraction, and buckling, while neglecting dynamics to focus on movement capabilities [17], [58].

The main approach has been to use a force balance derivation [146] to relate the actuation pressure to the robot length, bending angle, buckling threshold, growth force and cumulative resistance to eversion. A comprehensive review of this class of models was presented in [17]. The growth models were later extended to consider the working channels inside these robots [29], [160]. Alternatively, beam theory [83], [84], inflated beam models [51], [62], and the principle of virtual work (PVW) were employed [81], [125]. These models did not consider the internal pressure’s stiffening effect.

Tuctu *et al.* [62] employed constant curvature kinematic and inflated beam bending models, updated iteratively for deformations. The robot’s geometry results were integrated with a quasi-static model to accurately predict tip deflection. A similar method was employed by Hwee *et al.* [51] to model the robot displacement and curvature under external contact and load. Beam theory has also been employed to model magnetic steering [28], [88], [89], and low melting point alloy-based stiffening [104] mechanisms for eversion growing robots.

### C. Dynamics

Pressure changes or external impulses can cause rapid growth and bending in growing robots, necessitating dynamic models. These models mainly apply to everting robots, as tip-material deposition robots typically don’t exhibit rapid effects.

El-Hussieny *et al.* [129] used constant curvature kinematics and assumed all mass at the tip to present a dynamics model for everting robots using the Euler-Lagrange formalism. The model showed the coupling between bending angle and tip extension in various scenarios. Jitosho *et al.* [136] presented a dynamic simulator using an impulse-velocity formulation that assumed the everting robot was a series of rigid prismatic joints and links. This model was also defined using the Lagrange multiplier formulation. A similar framework based on differentiable simulation, which enables the implementation of the model within gradient-based optimization approaches, is presented in [154]. This framework integrates a closed-form nonlinear stiffness model for thin-walled inflated tubes to capture material wrinkling. Both simulators are open-source.

The model of [22], using the TMT dynamic method from [161] for derivations, was capable of capturing eversion dynamics by modeling the stationary and sliding parts of an everting robot as a pair of length-varying concentric tubes. Extended dynamic modeling has been also presented beyond the growing structure by incorporating actuation, specifically system pressure dynamics. Such models were utilized for friction-assisted growth, where frictional contact with an internal structure, such as a catheter, aids in the robot’s eversion [153], and growth length control [115].

### D. Finite Element Modeling

Although experimentally studied, the limiting factors on the scale and performance of growing robots are not fully known, such as the impact of everting geometry on internal friction. High-fidelity Finite Element Analysis (FEA) models have provided insights into these issues. Wu *et al.* [44] developed the first physics-based model for tendon-driven growing robots, considering internal pressure, tip force, and tendon tension effects on stiffness, growth speed, and steering. Implemented in the SOFA framework [162], [163], the model showed low accuracy compared to experiments due to its simplicity. A more accurate eversion sheath model was presented in [156], using Cosserat rod strands to capture various tip eversion patterns. However, high computational cost and simulation instability at larger time steps and velocities remain challenges.

## VI. Control and Planning

This section reviews the path planning and control methods for growing robots, see Table II and Supplementary-Figure 2.

### A. Path Planning and Environment Mapping

Sliding-free motion and compliance of everting growing robots allow effective use of environmental contacts for steering and path planning. Greer *et al.* [71], [137] introduced this concept for a non-steerable robot, using a recursive 2D path planning method to adjust robot length and base direction for desired tip contact angles with known obstacles. A similar technique was applied to additive manufacturing-based growing robots to study 3D maneuverability and system workspace [127]. Selvaggio *et al.* [144] evolved this into steerable, underactuated everting robot with controllable bending angles.

When local bending and shape setting is possible, e.g. via heat welding or tip tendon steering, standard path planning techniques such as Rapidly-exploring Random Tree star (RRT*) algorithms [99] and node-based A-start (A*) techniques enabled by real-time imaging [135] can be used. The development of more effective local steering can enable further implementation of similar standard techniques in the future, especially for contact-sensitive medical applications. Finally, Fuentes *et al.* [164] demonstrated that in the absence of prior environmental knowledge, a robot’s interaction along its deployed length can be used for mapping.

### B. Model-based Control and Observation

Compensating for model uncertainties with adaptive terms can enhance closed-loop control of growing robots by improving transient response, avoiding overshoots, and accounting for disturbances (e.g., pressure changes and external forces) [84], [155]. To this end, Ataka *et al.* [83] introduced the first model-based controller for fPAM-actuated everting robots, considering their ability to change structural stiffness using pressure. They extended this work in [84] to estimate unknown parameters using a Kalman filter-based observer, which utilized pressure and bending sensor data to control position and orientation with improved accuracy. El-Hussieny *et al.* [131] applied a non-linear model predictive control scheme with Monte-Carlo simulation to enhance controller robustness and guide growth direction. Energy-shaping [155] and high-order sliding-mode [115] controllers based on a 1D model were presented that used a Hamiltonian formulation for ideal gas and a nonlinear observer to take into account detailed pneumatic actuation mechanics, e.g. actuation medium pressure-density relation, as well as external disturbances. More recently, Wu *et al.* [125] introduced a switching controller combining model-based open-loop (using Constant Curvature kinematics and PVW mechanics) for coarse motions and model-free proportional closed-loop control for fine motions.

### C. Data-Driven and Model-Free Control

While model-based techniques offer insights into structural and controller design implications, data-driven methods can provide higher accuracy and competitive computational performance for complex systems [165].

Watson *et al.* [157] used real-time position and orientation data to update the robot kinematics model via closed-loop position control with Jacobian corrections, enabling autonomous tip localization and position control. However, this approach depended on sensing quality and was limited to slowly-moving robots with minimal external disturbances.

Some studies have explored model-free methods for controlling growing robots. AlAttar *et al.* [145] approximated the system model with a volatile local linear model, evaluating a closed-loop controller in simulations. Deep reinforcement learning framework were explored based on Proximal Policy Optimization [158] and reward shaping strategy [159] that adapted to the increasing length and hence the degrees-of-freedom of a growing robot. The former approach illustrated advantages over a Jacobian-based controller in 2D simulations. Finally, El-Hussieny *et al.* [134] introduced a deep Reinforcement Learning (RL) framework for obstacle-aware control.

Overall, data-driven techniques can handle modeling uncertainties, but their usability is impacted by unmodeled system dynamics and environmental interactions.

### D. Teleoperation and Autonomy

Teleoperation allows users to make decisions and interact with unknown environments in real-time during robot navigation. El-Hussieny *et al.* [59] introduced a flexible joystick that mapped human input into robot shapes and movements. An adaptation added a camera to the robot tip [85] for better situational awareness. However, control interfaces dissimilar to the robot’s kinematics require operators to learn complex mappings between the interface, actuator, and robot degrees-of-freedom. Stroppa *et al.* [138] proposed motion tracking with tactile and haptic feedback to assist operators. Teleoperation is the first step towards semi or fully autonomous systems.

Closed-loop strategies update models based on sensor data [157], enabling autonomous position control without line-of-sight. However, autonomous control relies on sensor quality, as poor data can distort the system. Autonomous self-growth, driven by reactive behavior in response to stimuli, is emerging as a key approach for navigating and exploring unstructured environments [15]. Tip-mounted sensors can provide feedback for directional growth, inspired by plant root chemically-guided growth [166]. Measurement systems help robots adapt their shape and respond to changes [167], but fully autonomous systems depend heavily on algorithmic interpretation of sensor signals.

Semi-autonomous schemes balance human teleoperation and algorithmic perception, allowing humans to handle complex tasks while reducing cognitive load [138], such as controlling only a subset of the robot’s degrees-of-freedom.

## VII. Applications

**Figure 5.**
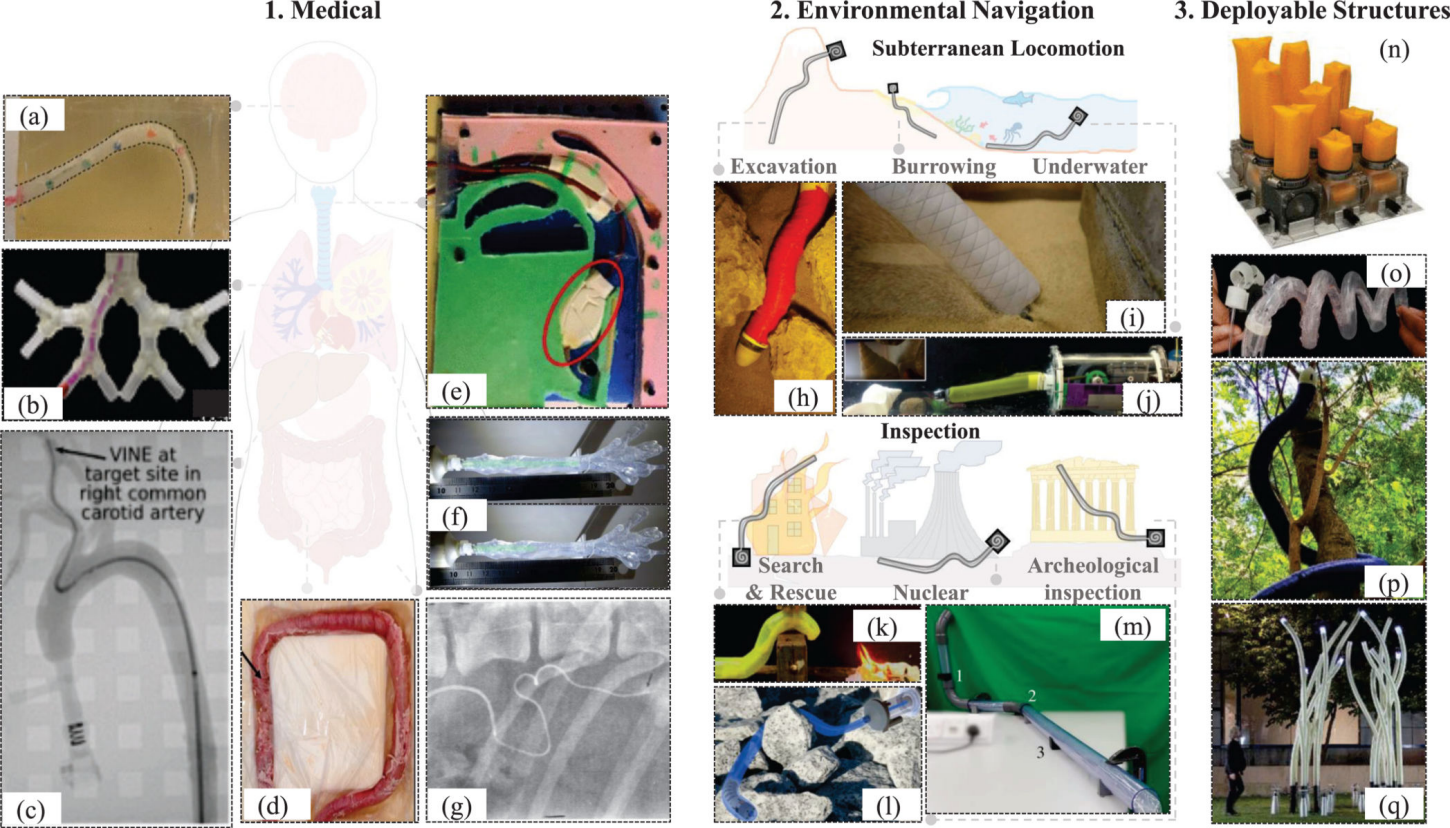
Growing robots are observed under three main application categories. 1. Medical applications: **(a)** soft catheter for neuro-interventional surgery [73], **(b)** navigating through the respiratory system [31], **(c)** VINE catheter for endovascular surgery [72], **(d)** colonoscopy robot [61], **(e)** emergency airway device [90], **(f)** MAMMOBOT to detect early-stage breast cancer [22] and **(g)** intraluminal navigation through blood vessels in a dog [168]. 2. Environmental navigation, including subterranean locomotion e.g. burrowing, as in **(h)** [14] and **(i)** [126], or **(j)** underwater robots to explore coral reefs [30], or inspection, such as **(k)** detecting and extinguishing a fire [11], **(l)** navigating through rubble [93], and **(m)** radiation monitoring [169]. 3. Deployable structures, e.g. **(n)** soft growing pins array [151], **(o)** wearable haptics [23], **(p)** climbing and twining robot via additive manufacturing [15], and (q) architectural structures [39]

**Figure 6.**
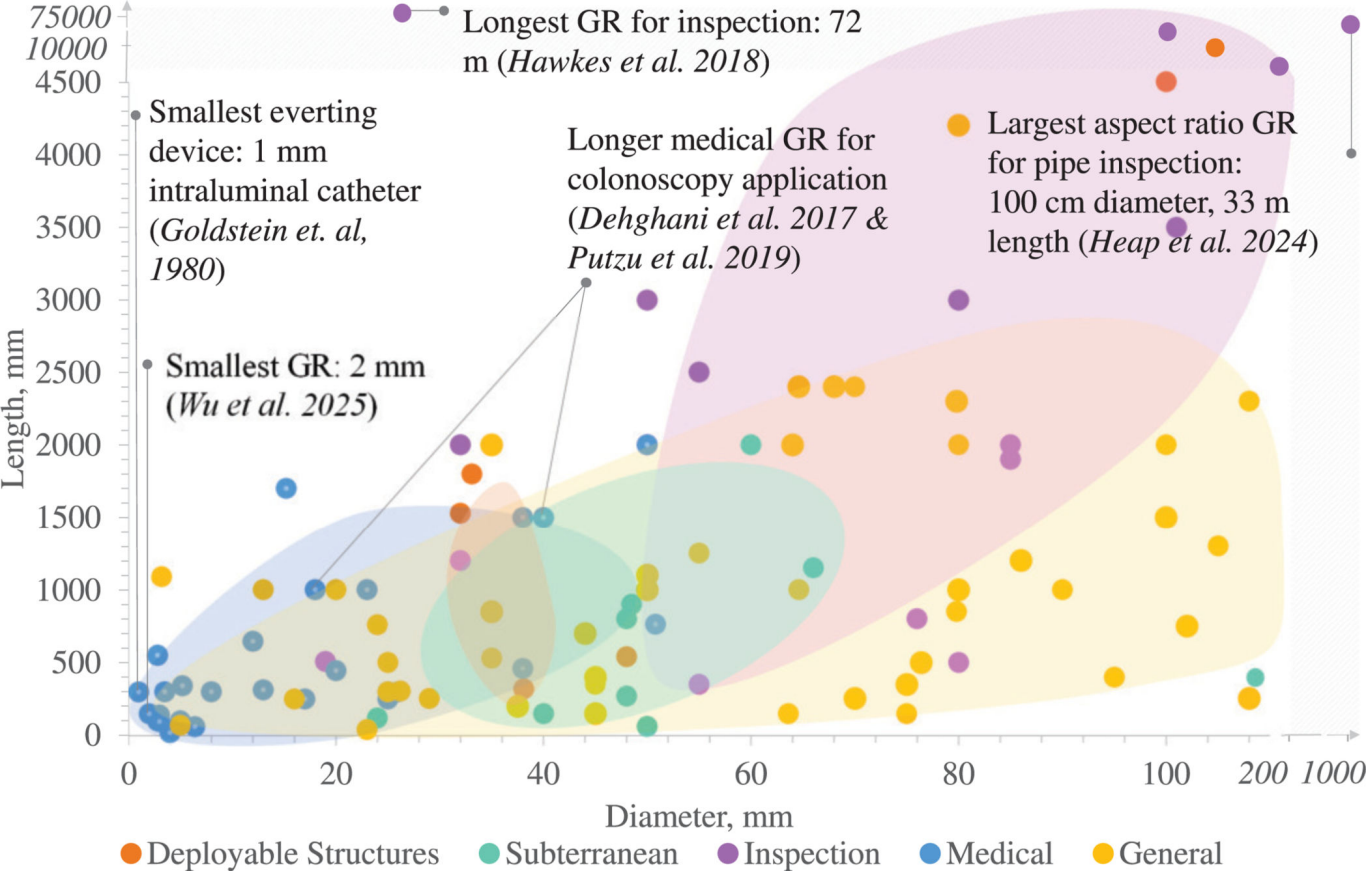
Dimension trends in Growing Robots (GR) for applications: deployable structures, subterranean navigation, inspection, medical, and general.

The inherent material properties and method of tip-growth make such robots ideal for deployment in torturous, constrained environments. They are well-suited for tasks like reaching subterranean levels, searching for rescue victims, navigating complex human anatomies in minimally invasive surgeries, and constructing deployable structures, such as antennas and architectural frameworks. Figure 5 summarizes the different applications of growing robots, and Figure 6 highlights how the robot dimensions heavily depend on the application.

### A. Environmental Navigation

- **Search-and-Rescue and Industrial Inspection:** The earliest eversion robots were aimed towards inspection applications, when Mishima *et al.* [9] identified their low external friction and flexibility, which made them ideal for navigating torturous terrain and rubble. Tsukagoshi *et al.* [10] proposed to steer via two parallel tubes integrated inside the robot to navigate through rubble. Growing robots have since been used for tasks such as pipe inspection [45], archaeological exploration [85], rescuing trapped victims [27], [86], [170], and navigating, sensing and decontaminating nuclear environments [94], [169].
  As indicated in Figure 6, inspection robots typically have the largest dimensions, with lengths extending up to 72 m [11] and diameters as large as 1 m [45]. Hence, they are predominantly elongated via eversion, as the deployment of additive manufacturing-based growing robots is restricted by the 3D printer’s operational limits, e.g. printing speed and time, power consumption, overheating.
- **Subterranean:** Burrowing robots, inspired by plant roots and animals that penetrate soil by reducing the drag forces, often combine tip eversion with complementary techniques like granular fluidization and asymmetric tip shapes to enable efficient burrowing [126], [147]. As discussed, the “plantoid” [13], which grows through additive manufacturing, was developed for subterranean tasks. It exploited the viscoelastic properties of PLA to escape obstacles [14]. Another subterranean application is excavation, as in the RootBot [41]. Similar to early patented trenchless piping eversion tubes, the RootBot comprised two everting tubes connected to an excavation module to enhance their steering and retraction capabilities while enabling directional excavation. A discharge module was incorporated to prevent excavated soils from accumulating.

Underwater applications, such as navigation through coral reefs, also benefit from eversion robots. In [171], it was indicated that tip growth velocity increases with water flow rate, but is inversely proportional to the depth. An added requirement for underwater applications is buoyancy, allowing the robot to float and extend to great lengths without buckling. In [30], [82], water, the same fluid as the surrounding environment, was used as the actuation fluid to make achieve neutral buoyancy. This concept was utilized in [82] to hydraulically inflate bending pouches via volume change, enabling steering in underwater applications. Conversely, Kaleel *et al.* [140] suggested the use of helium as the pressurization medium to develop a buoyancy control model for a floating eversion robot in air, and later made use of the concept in underwater systems.

### B. Medical

Tip growing robots have the potential to increase patient safety and minimize tissue damage. Medically-relevant growing robots have so far only utilized eversion due to scaling constraints and safety considerations. They are commonly miniaturized, with diameters averaging 19.9 mm (see Figure 6), to fit through small anatomical openings.

Eversion robots particularly attractive in applications such as endoscopy [61], [98], [115], [177]–[179], where they help manage the friction between the device and the organ wall. Shike *et al.* [180] integrated an everting sleeve onto traditional colonoscopes, reducing shear forces during growth. In [61], a growing robot was used as the colonoscope itself, with an inflated latex tube enabling navigation through the colon to a lengths of 1.5 m, matching the colon’s average length. Although it conformed to the colon path more easily than conventional tools, it still required skilled manipulation. Saxena *et al.* [178] automated steering to reduce skill requirements and procedure time. Eversion movements in colonoscopy also need to be accurately controlled such that they are smooth and steady, enabling high quality image acquisition and visualization of important anatomical details [115]. While previous designs demonstrated successful insertion and navigation through the colon, retraction [61], [178] or tool passing [61], [115], [178] were not possible. In [29], a tip-scrunching mechanism was integrated into a colonoscopy robot for tool passage.

Growing robots have also been used for airway access [88], [90], [181], where Hwee *et al.* [90] designed a dual-balloon system that sealed either airway (trachea or esophagus) and provided pulmonary ventilation to the alternative airway via a tube passing through the robot. This functioned in a similar way to endotracheal tube cuffs, but without the associated insertion and frictional forces.

In addition, growing robots can address challenges of catheter insertion and manual-steering during endovascular [72], spinal [46], and neuro-interventions [73], and navigating small branching cavities within the body organs such as the breast [22], [44], [125] and lungs [88], [89]. These robots can be pre-formed or actively steered via tendon-driven or magnetic mechanisms. Benchtop navigation tests indicated that growing catheters have the potential to lead to safer, more efficient procedures than conventional catheters [72]. Wu *et al.* [46] demonstrated that global forces exerted during robotic catheter insertion were significantly higher than those of an eversion robot when navigating a spinal cord phantom. Moreover, growing catheters showed the capability of achieving sharper bends, desirable when navigating complex pathways, e.g. the brain ventricles [73], aorta [72] and ductal tree branches [22].

#### C. Deployable Structures

Growing robot deployable structures, inspired by biological processes and origami designs, unfold and transport packages into open space. They are reconfigurable and can adapt their shape based on environmental needs.

For example, physical shape change and reconfiguration can significantly enhance the performance of antennas. The everting antenna design in [33], [78] geometrically reconfigured itself based on frequency feedback, changing shape and angle without requiring structural support. Fuentes *et al.* [103] proposed creating deployable structures by permanently stiffening the robot at arbitrary points to facilitate shape change and increase payload capacity. Eversion structures can also be utilized for haptic interfaces, for instance using an array of soft growing pins [151], or wearable haptics [23].

Additive-manufacturing growing robots can often self-sustain, supporting their body weight through materials like interwoven fiberglass [39] or PLA deposition [15]. These robots have been used in large-scale architectural structures, e.g. bridges and walls [39], and for plant-like climbing in unstructured environments [15].

#### D. Industrial Adoption & Commercial Translation

The demand for trenchless piping methods in the 1970s, allowing pipeline repair with minimal excavation, led Wood *et al.* [4] to propose inserting a flattened tubular lining into a passageway via eversion. This technique evolved into inserting and curing resin linings through fluid pressure [4], [182], and placing lining pipes in conduits [183] via bladder eversion [184]. The success of eversion in piping and medical devices led to research and patents, focused on the functionalization of such mechanisms, e.g. via sensor integration, for nondestructive and safe navigation [173]–[175], [185]–[190]. Industrial interest is growing, especially in surgical technologies like “BreathFirst” everting airway device (Vine Devices Inc.) [181], [191], and in industrial pipe inspection [45].

**Figure 7.**
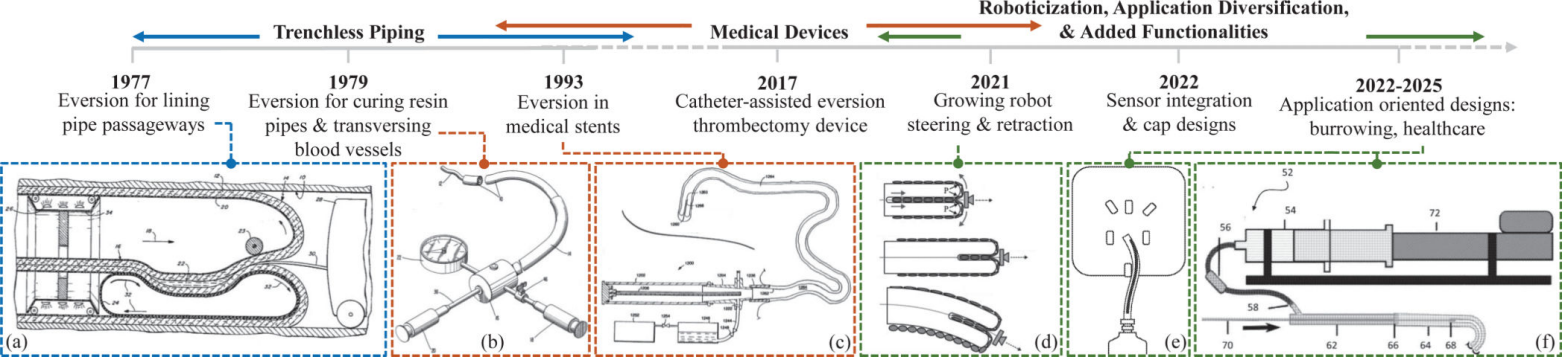
Timeline of patents demonstrating eversion mechanisms, first introduced by Wood *et al.* in 1977 for lining pipe passageways, as in **(a)** [4]. The eversion mechanism was then embedded into minimally invasive surgical devices, as in **(b)** [172]. Roboticized eversion was first patented in 2021 to showcase robot growth **(c)** [173]) and retraction **(d)** [174]. This was further developed by integrating sensors into the system. Application-oriented patents, such as **(e)** duty-cycle controller for deployment in the mammary duct [175] and **(f)** the VINE catheter [176], were demonstrated from 2021–2024.

Figure 7 maps the evolving landscape of eversion mechanisms across diverse domains — trenchless piping, medical technologies and functionalization. While patents provide valuable insights, they do not cover the entire research landscape, given the lengthy patenting process and that new applications or designs are not always patented. Furthermore, this trajectory is not strictly linear — the domains overlap and re-emerge in new contexts. For instance, while piping and intraluminal interventions differ in specific goals and environments, both share the challenge of accessing long, tight tubes, enabling cross-domain adaptation and exchange of eversion techniques. Notably, early eversion methods developed for piping influenced catheter designs, showing knowledge transfer. Further interdisciplinary transfer is evident through evolving features within the piping domain, which now incorporate additional functional capabilities [4], [45].

**Figure 8.**
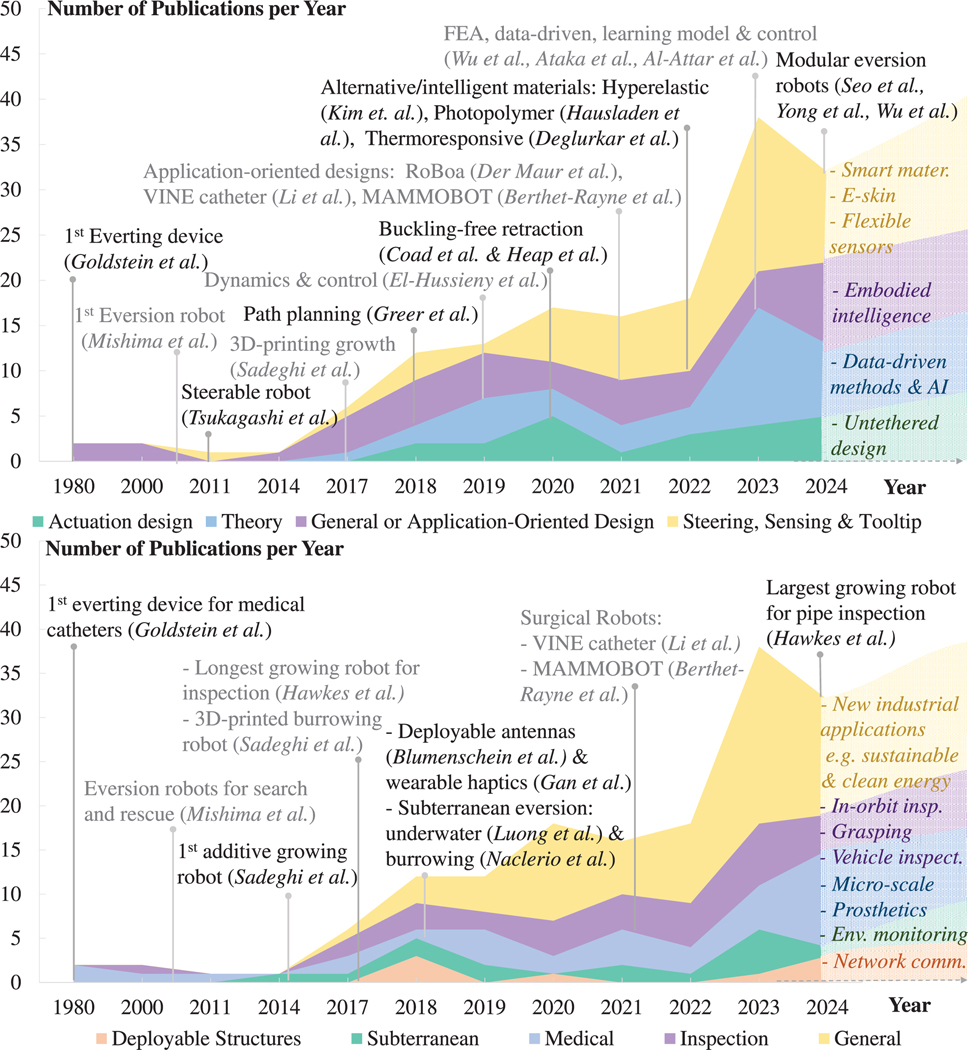
Number of publications on growing robot-related research by year, categorized by publication type (top) and application (bottom).

Building on these advancements, there has been a shift towards task-specific functionalities — complex path navigation, high-curvature bending and obstacle navigation — driven by fundamental progress and technological maturity of ‘growth’ mechanics, steering, sensing, and stiffening. The focus on function enables deployment in practical applications (see Figure 8), which overlap and lead to the revival of earlier concepts due to shifts in funding, evolving societal needs, and changes in interest areas.

## VIII. Discussions and Future Directions

Research on growing robots has surged since its reestablishment in 2017, with 150 papers and over 15 patents published (see Figure 8). A significant increase in publications has been observed since 2022, indicating a growing interest in the field. Early research primarily focused on application development, laying the foundation for growing robot design. In recent years, the focus has shifted towards steering, sensing, and theoretical investigations, furthering the understanding of growing robots. Nevertheless, the field remains at its infancy, with many open avenues for future research.

### A. Working Principle and Fabrication

Eversion robots typically use thermoplastic LDPE and ripstop nylon fabric for their ease of manufacturing and durability. The shift towards self-healing polymers and smart materials capable of responding to environmental changes shows promise in integrating multiple functionalities, such as sensing and steering, into the robot body. Self-healing polymers could enhance the durability of growing robots by repairing tears that would otherwise lead to failure, thus improving the robustness and longevity of the system. Programmable materials that regenerate by changing their structure or extending apically are promising for both eversion and additive growing robots.

While various fabrication methods have been explored, there remains a need for more automated processes to ensure robustness and repeatability. Recent advances in laser and ultrasonic welding show potential in automating fabrication. Additive growing robots also face material optimization challenges, balancing factors like melting points for stability, stiffness and growth speed. Multi-material FDM-printing could address these demands at different stages of robot fabrication.

Pressure-driven eversion and additive manufacturing actuation mechanisms distinguish growing robots from other soft robots but also pose significant challenges. Growing robots are tethered to their base, requiring large volumes of material for long distance travel. This issue is exacerbated in eversion robots, as growth length is constrained by pressure. Investigating designs where robots recycle their own material, similar to everting toroidal robots, or utilize material from their external environment for elongation, could yield attractive solutions.

### B. Steering, State Change and Retraction

Steering methods have explored both passive and active approaches. Passive steering largely focused on pre-defined and contact-based steering, which are effective but limited to specific environments. Combining these with smart materials and improved fabrication could enhance passive steering performance. Active distributed steering is limited by its integration into the robot body, affecting miniaturization. Tendon-driven steering also needs optimization to avoid interference with eversion. Similarly, active local steering often requires rigid elements, limiting compliance and scalability.

In a 2D environment, the inherent compliance of eversion robots is not a limitation, but in 3D environments, the suspension of the robot body can lead to mechanically self-loading, potentially causing the robot to collapse. Additive manufacturing robots are less compliant but require architectures and materials capable of supporting increased robot weight. Variable stiffness and shape-locking mechanisms could provide stronger mechanical support on demand, by transitioning to a rigid state while preserving compliance at desired points. Many state-changing eversion robots require additional hardware or material integrated into the robot’s body, affecting eversion performance and scalability. Recent efforts to functionalize the working fluid for both stiffening and pressurization show promise but limit tip growth to the compliant state. Exploring shape memory alloys, granular jamming, or hardware mechanisms that do not occupy space along the body could advance state change in eversion robots.

Finally, while there has been significant work on retracting growing robots, the focus was on eversion growing robots. As a result, 3D-printed growing robots still lack a retraction mechanism, limiting their deployment in environments where is retraction required. In [15], it was proposed that as an alternative to retraction (for environments that do not necessitate reversible growth solutions), biocompatible filaments, such as the PCL/SA filaments used in [69], can be explored in the future to grow and retain structures as long-term, permanent solutions in the same location as they were initially deployed. Another suggestion to remove such structures from their environments could be to use soluble filaments, such as PVA which can be dissolved entirely when exposed to water.

### C. Sensing and Perception

A key challenge in growing robots is sensor integration for tip and distributed sensing. Tip-mounted sensors are rigid, adding weight and limiting the aperture size the robot can grow through, with issues like buckling and friction remaining problematic. Challenges with distributed sensing hindered progress with proprioceptive sensing, such as shape and curvature tracking. Recent advancements have distributed and integrated sensors directly into steering pouches, reducing external friction. Miniaturized and flexible sensors that can evert with the robot body will advance internal sensor integration. This also pushes towards the development of sensorless tracking mechanisms. For instance, magnetic skin has been used for steering eversion robots [88], opening up the possibility for sensorless tip localization and shape sensing by tracking changes in the external magnetic field as the robot grows. This can be extended to additive growing robots, using magnetic printing filament. Moreover, growing robots undergo shape changes upon contact with obstacles and length changes during apical extension, leading to deformations in the robot body. In eversion robots, pressure-based deformation tracking was previously deployed to sense contact forces [119]. This can be applied to other sensing mechanisms, such as tip localization, making use of the relationship between the feeding pressure and eversion length. Rather than integrating pressure sensors onboard, they can be positioned at the base as in to infer localization [114], contact and shape information. Pressure, deformation and curvature changes can be used with AI-based, model-based or state-estimation-based sensing, as explored in [115] for tip localization.

### D. Modeling, Control, & Autonomy

The complexity of the underlying actuation, steering, and state change mechanisms for growing robots as well as the growing motion in realistic scenarios with environmental interactions necessitate high-fidelity, scalable and computationally efficient models. Future directions on modeling and control of growing robots can be inspired by recent relevant developments within the Soft Robotics community [192] on reduced-order [193], model-based [194], and learning-based [165] control frameworks as well as high fidelity Finite Element techniques. Further development of open-source simulation toolboxes for growing robots, such as those presented in [22], [136], [145], [156], will also facilitate the utilization of advanced dynamic modeling and control techniques for growing robots autonomy, and the creation of reliable benchmarks to compare the variety of developed techniques.

### E. Applications

Inspection tasks have been the primary application of growing robots. Designing deployable structures, such as antennas, could enable the use of growing robots in wireless network applications. Attention is also drawn to maintenance and inspection of tight spaces, e.g. in-engine, in-orbit applications, grasping, and architecture applications.

Subterranean robots have incorporated novel techniques to overcome resistive forces of burrowing and excavation. Subterranean robots were also shown to support sustainability and address climate change challenges, both underground by monitoring soil conditions, and underwater by exploring the conditions needed for sustaining coral reefs. Overcoming resistive forces while navigating through soil, sand or water, however, remain challenging. As suggested in [65], further bioinspiration from plant root growth can advance the soil penetration performance of subterranean growing robots by implementing features such as circumnutation and radial expansion. Similarly, achieving controllable buoyancy in underwater robots is a challenge to be solved.

At a smaller scale, medically-relevant robots hold the potential to reduce patient discomfort and increase safety. However, miniaturization remains a challenge, limiting the anatomies in which they can be deployed. This limitation arises from constraints in fabrication techniques, pressure requirements and the need to maintain flexibility. Exploring new structural designs and alternative materials that do not compromise adaptability is crucial. While the is currently on minimally invasive surgery, the recent demonstration of grasping in [118], combined with their ability to grow and retract, suggests potential applications in limb prosthetics.

### F. Alternative, Emerging Concepts for Tip-Growing Robots

Growing robots face limitations in load-bearing capacity due to their materials and composition. Eversion robots, in particular, lack a base to mount an end-effector, and precise positioning is challenging. Yan *et al.* [19] introduced a chain-block system that grows by transporting fluidized material to the tip, where it solidifies and self-supports, increasing load-bearing capacity. Additionally, growing robots typically feature a fixed base with only the tip moving. Quan *et al.* [20] presented a tape-measure growing robot with a wheeled base that drives along the path, using magnetic grippers to anchor onto and climb surfaces, overcoming length limitations of climbing and growing robots.

Further inspiration can be drawn from biological growth and pressure-exchange processes. Growing robots require a reservoir of material, are always tethered to the base, and in the case of eversion robots, require a constant pressure supply for growth. A key question is: how can we harness the surrounding environment to address these challenges?

Algae-based PLA and seawater cement have been explored for 3D printing, making in-situ underwater fabrication a possible research direction for additive growth, and similar methods can be applied to subterranean or space environments. Osmotic pressure exchange processes could inspire hydraulic eversion robots that absorb pressure/fluid from their surroundings to continue growing, overcoming the pressure limitations of current designs and enabling the creation of smaller, longer and more robust eversion robots. Biological processes like DNA-origami and reassembly could facilitate shape morphing and self-assembly in future tip-growing robots.

## IX. Conclusion

Growing robots navigate complex and constrained environments via pressure-driven eversion or additive manufacturing. They deposit new material at the tip, allowing them to extend to greater lengths than traditional continuum robots. Their inherent compliance, use of entirely soft materials, and unique locomotion strategies make them well-suited for highly versatile environments. In this review, we highlighted the design, steering, sensing, and control strategies used in growing robots, as well as their main application areas to date. The features and limitations of existing robot designs and models are provided, with the expectation of seeking new approaches to tackle such challenges.

Growing robots enjoy a renewed interest in the field of soft robotics, leveraging features from early research since the 1980s in the piping and medical industries, and taking bioinspiration from plants in the surrounding environment. Thanks to their unique properties and presented challenges, they represent a promising area for future research.

By building on the knowledge and foundation for new growing robot technologies that has been laid by research to date, there is strong potential for the real-world deployment of these robots. With further investigation in the upcoming years, the research community can help solve the unanswered questions and key challenges currently hindering progress in order to increase the impact of soft growing robots in society.

## Supplementary Material

supp1-3608701

## Figures and Tables

**Table I:** Steering Mechanisms in Growing Robots

Mechanism and Methods	Advantages (+) and Limitations (−)
**Passive Steering:**	
- Pre-defined [73]	+ Simple; no actuators needed for steering − Not-steerable on-demand; path should be known
- Contact-based: Passive morphological adaptation [14], [90]	+ Simple deployment, no additional hardware required − Not deployable in open environments or those with multiple paths; reachable path is limited by obstacle arrangement; high shear forces against the environment
Passive adaptation by external stimuli	
- PPSA* [75]	+ Material-level responsiveness; instant response − Deployment only in open environments
**Active Steering:**	
Distributed steering	
- Tendon-driven [22]	+ Simple modeling and control; constant curvature bend − Tendon-routing friction; non-uniform curvature
- Pneumatic Artificial Muscle (PAM*): SPM* [84], sPAM* [80], fPAM* [54], iPAM* [33]	+ Entirely soft design, minimal friction; variable stiffness with fPAM − Non-linearity for stretchable fabric; limited steerability due to serial connection; can occupy large volumes
- Magnetic [88]	+ Scalable; embedded as the robot skin or as actuation fluid − Requires close magnet-robot proximity; magnetic skin repeatability limited by bonds between robot layers
Localized steering	
- Latches [11]	+ Entirely soft actuator; simple fabrication and control − Irreversible
- Magnetic valves for cPAM* [91]	+ Selective pouch steering; highly bendable; high lateral forces − Large volume with slow response
- Internal device: rigid articulated links [92], TSA* [93], continuum robot [94], reeling [95], heat welding [96]	+ High payload; sharp and precise bends at arbitrary points; often facilitates buckling-free retraction − Heavy; rigid components limit compliance e.g. for passing narrow openings
- Additive: e.g. FDM* [15]	+ Fully-autonomous; variable stiffness; speedy growth; self weight support; 3D steering − Slow; irreversible; not retractable

* **PPSA**: photothermal phase-change series actuator; **SPM**: series pouch motor; **PAM**: pneumatic artificial muscles; **sPAM**: series PAM; **fPAM**: fabric PAM; **iPAM**: inverse PAM; **cPAM**: cylindrical PAM; **TSA**: twisted string actuator; **FDM**: fused deposition modeling printing.

**Table II:** Modeling, path planning, & control methods.

Domain / Method (% share of literature)	Study type^1^ (error%)	Planning & control methods	Features (*software*)

**Analytical (94%)**			
- Kinematics (33%)			
Const. Curv. (17%) [15], [31], [34], [59], [60], [62], [66], [76], [83]–[85], [91], [98], [99], [108], [125], [127]–[135]	SE-2,3D (2–20% model, 0.5–3% control)	Switching, Jacobianbased, Force, Planing, Predictive, Moving horizon, Teleoperation	Simplicity
PRB* (8%) [71], [95], [106], [117], [136]–[141]	SE-2,3D (5-6% pos., 5% angle)	Path planning, Contact obs., Shared-control	Design opt. (*Vine_Simulator*)
Variable Curv. [33], [79]	SE-3D (5%)	ND	Design opt.
Fitting^2^ [22], [113], [142]	SE-3D (1.4–5%)	ND	(*TMTDyn*)
Others^3^ [15], [26], [44], [75], [101], [139], [143]–[145]	SE-2,3D (ND)	Obstacle-aided, closed-loop, Jacobian, reactive	Design opt. (*SoroSim*)

- Quasi-statics (50%)			
Force balance (37%) [21], [32], [48], [58], [61], [63], [75], [83], [85], [93], [95], [100], [103], [107], [108], [110], [111], [146]–[148], [10], [11], [27]–[29], [34], [36], [38], [45], [55], [76], [78], [94], [101], [126], [149]–[152]	SE-2,3D (6% pos., 5% angle)	Force & magnetic position/ orientation control	Buckling, thermal model, burrowing
Beam (Inflated) [51], [62], [74], [83], [84], [88], [89], [104], [119]	SE-2,3D (15-20% tip pos., 6-9% force pos.)	Jacobian inv., Force localization, Observer	Accurate actuator model
PVW* [81], [125]	SE-2D (0.7-1.1%)	Switching closed-loop	Suitable for complex systems
Cosserat rod [144]	SE-2D (1.3%)	Obstacle-aided	Accurate

- Dynamics (12%)			
Lagrangian, Lumped par. [129], [130], [136], [153], [154]	SE-1,2D (4% growth velocity)	ND	Simple, pressure dyn. (*Vine_Simulator, DiffVineSimPy*)
Inv. dyn., PVC* [145]	S-3D (ND)	Jacobian controller	(*SoroSim*)
Hamiltonian, PVW* [22], [115], [155]	SE-1,3D (0.9-4.4%)	Energy shaping & sliding-mode controller	Physics-based, (*TMTDyn*)
FEA* [44], [156]	SE-1,3D (6–24% pos., 51% speed)	ND	Physics-based, (*SOFA*)

**Data-Driven (6%)**			
Jacobian^4^ [145], [157], [158]	SE-3D (0.7–4%)	Closed-loop	Accurate, efficient
Deep RL* [134], [158], [159]	S-2D (2–12%)	Trained controller	Accurate, adaptive


1 S/E: Simulation/Experimental, 1–3D: motion dimensionality, pos.: position, ND: No Data.

2 Shape function fitting: Piecewise Cubic Bezier, polynomial spline Curves.

3 Finite Element, trapezoidal mesh, Piecewise Variable Curvature, trigonometric relations, etc.

4 Jacobian correction.

* **PRB**: Pseudo Rigid Body (e.g. Joint-space representation & Prismatic-Revolute-Prismatic), **PVW**: Principle of Virtual Work, **PVC**: Piecewise Variable Curvature, **FEA**: Finite Element Analysis, **RL**: Reinforcement Learning.

## Footnotes

1
https://cgirerd.github.io/tip_growing_robots.html
