# Supplementary material for: Tip-Growing Robots: Design, Theory, Application
Source: IEEE Trans Robot. Author manuscript; Available in PMC 2026 Jan 17. (PMC12811027; doi:10.1109/tro.2025.3608701)
Supplement: supp1-3608701 [file NIHMS2114701-supplement-supp1-3608701.pdf]

Supplementary Material

Table I
MANUFACTURING METHODS TO CONSTRUCT THE GROWING ELEMENT.

**Mechanism (Material)**	**Features (+) & Limitations (-)**
**Welding:**	
Heat welding (thermoplastic films*, latex, silicone, TPU*-coated fabrics) [22]	+ Quick, low cost, high strength<br>- Thick weld, microfabrication issues
CO_2 Laser-welding (thermoplastic films) [49]	+ Repeatable, microscale, thin weld, bespoke shapes<br>- Low tear strength
US*-welding (TPU-coated fabrics, thermoplastic films) [50], [55]	+ Quick, efficient, repeatable, High strength<br>- Forms thick welds leading to microfabrication issues
**Adhesives:**	
Silicone adhesive (silicone-coated fabrics) [54]	+ Fast curing time, bespoke shapes<br>- Materials specific, low repeatability
Polyurethane adhesive (TPU, TPU-coated fabrics) [72]	+ Bespoke shapes<br>- Long curing time, low strength, low repeatability
Adhesive tape (fabrics, TPU, polypropylene) [50]	+ Quick, simple<br>- Microfabrication issues, low strength
**Stitching:**	
Sewing (fabrics) [94], [111]	+ Repeatable, quick, bespoke shapes, high strength<br>- Sealing & microfabrication issues
**Additive Manufacturing:**	
FDM-printing (PLA*, PCL*) [13]	+ Self-supporting, active stiffness modulation, multi-environment<br>- Short term reliability, limited scalability, limited use-cases due to printing speed & friction exertion
Weaving & UV*-curing (fiberglass) [39]	+ Durable, Self-weight support<br>- As FDM-printing
Photopolymerization (monomer solution) [40]	+ Easy transfer of material, active stiffness modulation<br>- No active steering, no inside body passage

*Thermoplastic films, e.g. LDPE, HDPE, TPU and polypropylene. **TPU**: Thermoplastic Polyurethane, **PLA**: polylactic Acid, **PCL**: Polycaprolactone, **LDPE**: Low Density Polyethylene, **HDPE**: High Density Polyethylene, **UV**: Ultra-Violet, **US**: Ultra-Sound.

Table II
VARIABLE STIFFNESS MECHANISMS DEPLOYED IN GROWING ROBOTS

**Mechanism**	**Advantages (+) and Limitations (-)**
Pneumatic expansion [60], [62], [73], [81], [83], [84], [105]	+ High stiffness ratio<br>- Limited by pouch geometry, limited accuracy at low pressures
Layer jamming [56], [102], [106], [107]	+ Simple deployment<br>- Limited efficacy, miniaturization issues
Heat-welding [58], [99]	+ Shape-locking, reversible variable stiffness<br>- Limited by the number of shape-locking bodies, irreversible steering
Foam-based [103]	+ Passive shape-locking<br>- Slow response, environmental damage upon solidification
Phase-changing medium [104]	+ High stiffness ratio, dual functionality as stiffening and actuation medium hence simple integration<br>- Slow response time, requires continuous heating, forms sharp edges when solidified

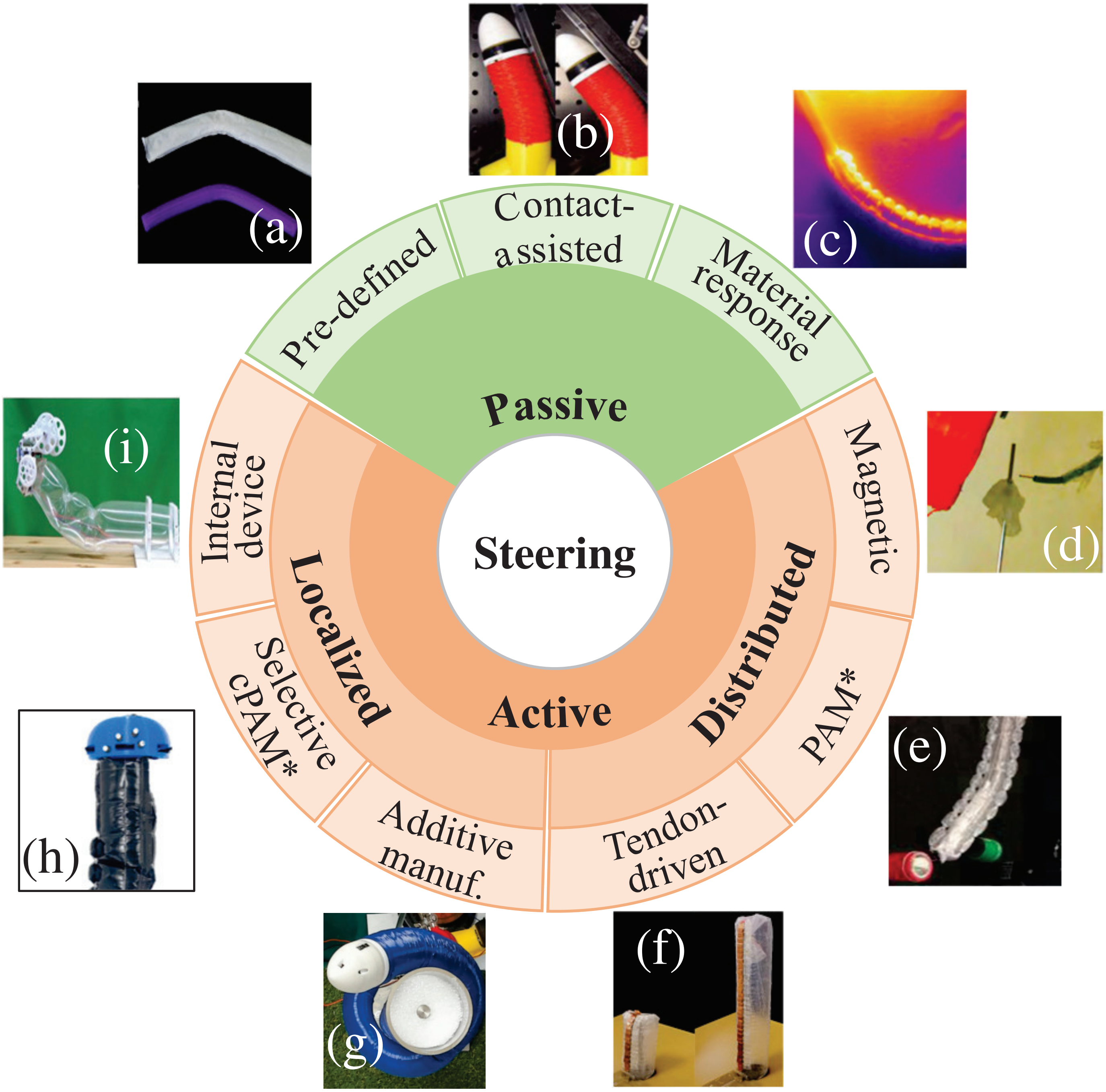

Figure 1. Steering mechanisms deployed in growing robots. **Passive steering:** (a) Pre-forming the robot body by molding and heat-sealing [73], (b) Contact-based steering, where PLA changes its viscoelastic properties to turn around obstacles [14], (c) Robot body-material response to light and heat [75]. **Active distributed steering:** (d) Magnetic [89], (e) Pneumatic Artificial Muscles (PAM) [80], (f) Tendon-driven [33]. **Active localized steering:** (g) Tuning printing parameters via additive-manufacturing [15] (h) Localized cPAM inflation [91] (i) Steering eversion robots via internal-device, e.g. adjusting path via real-time heat-welding [58]. *See Table I subscript.

Table III
SENSOR INTEGRATION & IMPLEMENTATION IN GROWING ROBOTS.

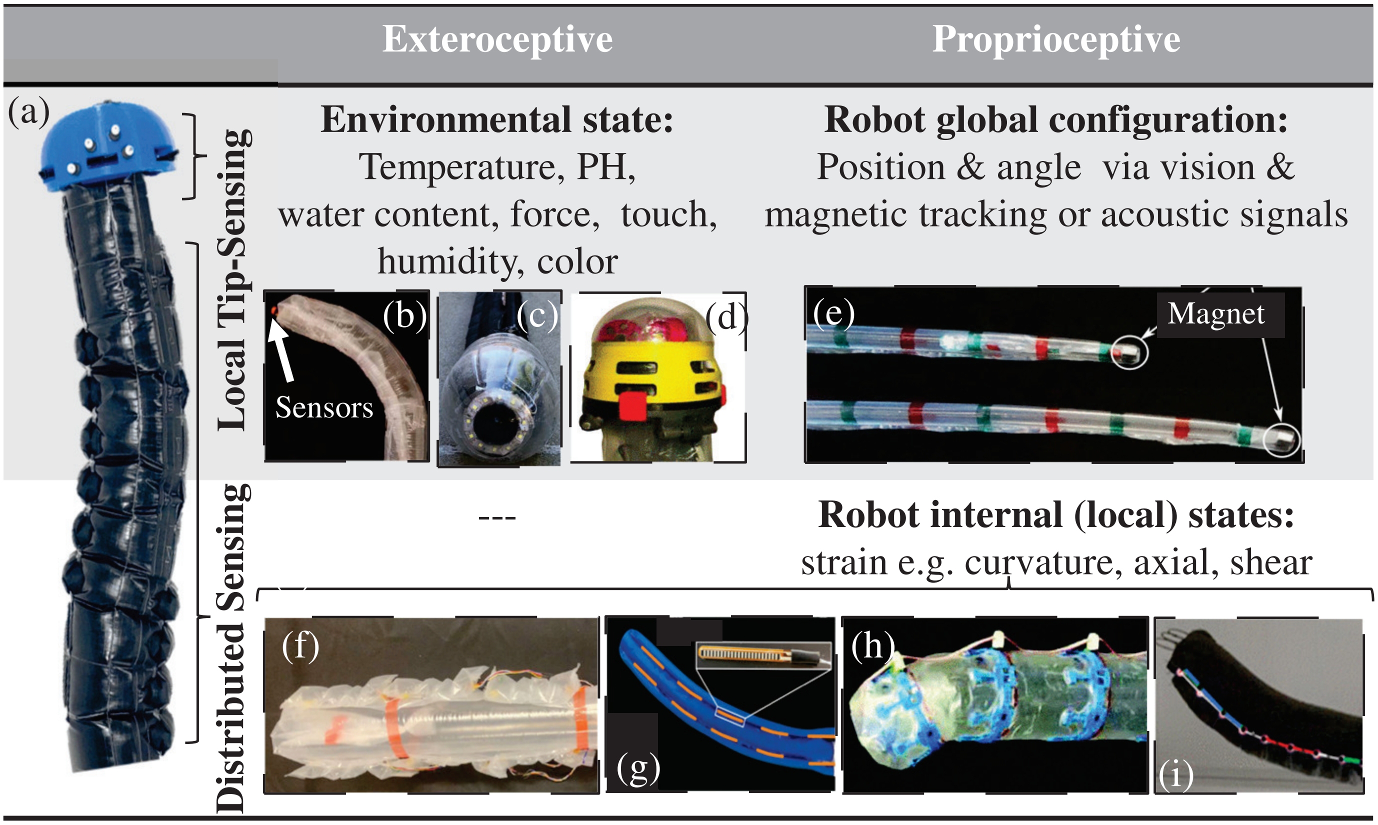

(a) Sensor integration [91] through 1. Exteroceptive sensing (always localized), e.g. to provide visual feedback via **(b)** string-mounted [11] or **(c)** cap-mounted camera [85], or **(d)** to measure soil conditions [42]. 2. Tip-localized proprioceptive sensing e.g. to capture the robot's position via **(e)** magnetic tracking [112]. 3. Distributed proprioceptive sensing to measure strain by **(f-g)** embedding sensors into pouches along the robot body [119], [120], **(h-i)** or along the exterior surface of robot [116], [118].

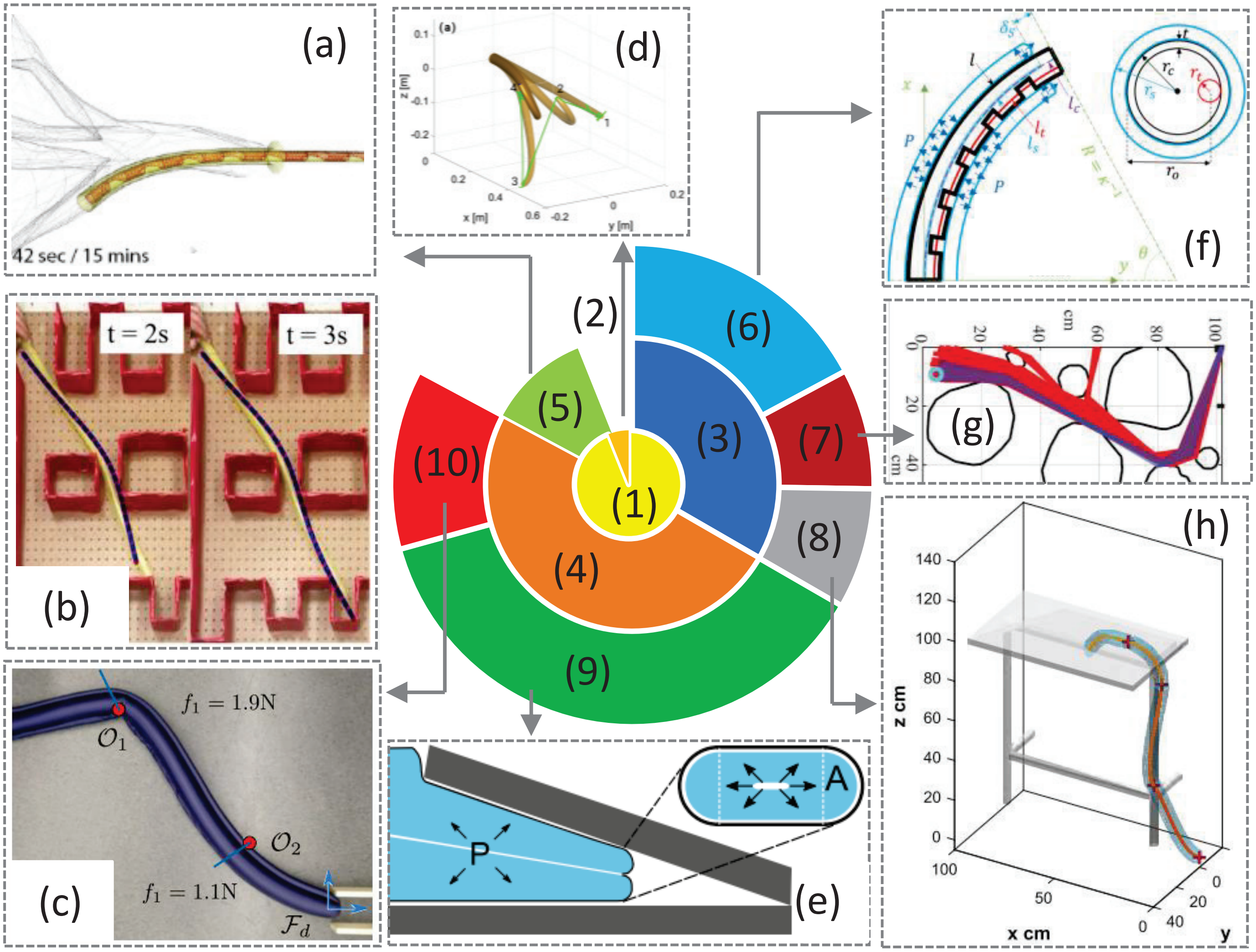

Figure 2. A piechart and snapshots of the different modeling techniques for growing robots in the literature: **(1)** Analytical 94%, **(2)** Data-driven 6%, **(3)** Kinematics-only 33%, **(4)** Quasi-statics 50%, **(5)** Dynamics 12%, **(6)** Constant Curvature 17%, **(7)** Pseudo Rigid Body (PRB) 8%, **(8)** Other kinematics 8%, **(9)** Force balance 37%, **(10)** Other quasi-static mechanics 12%. Analytical quasi-static modeling via force-balance principles is the most widely utilized framework. **(a)** Finite Element Analysis (FEA) using *SOFA-Framework* for physics-based eversion growth and environmental interactions [44] dynamics. **(b)** PRB dynamics implemented in *Vine_Simulator* software [136]; **(c)**. Quasi-static models based on Cosserat rod model for obstacle-interaction planning [144]. **(d)** Data-driven controllers tested in simulation (using *SoroSim* software) [145]. **(e)** force balance model for growth through a slanted gap [146]. **(f)** kinematic models based on Constant Curvature for closed-loop control [125]. **(g)** PRB for navigation by exploiting environmental contacts [71]. **(h)** Shape function approximation for robot geometric design [142].
